# Sensor Fusion and Smart Sensor in Sports and Biomedical Applications

**DOI:** 10.3390/s16101569

**Published:** 2016-09-23

**Authors:** José Jair Alves Mendes, Mário Elias Marinho Vieira, Marcelo Bissi Pires, Sergio Luiz Stevan

**Affiliations:** 1 Department of Electronic Engineering, Federal University of Technology of Parana (UTFPR), Ponta Grossa (PR) 84016-210, Brazil; josej@alunos.utfpr.edu.br (J.J.A.M.J.); mariovieira@alunos.utfpr.edu.br (M.E.M.V.); mpires@alunos.utfpr.edu.br (M.B.P.); 2 Graduate Program in Electrical Engineering (PPGEE), Federal University of Technology of Parana (UTFPR), Ponta Grossa (PR) 84016-210, Brazil

**Keywords:** smart sensors, sensor fusion, biomedical, sports, rehabilitation, development of performance in athletes, J0101

## Abstract

The following work presents an overview of smart sensors and sensor fusion targeted at biomedical applications and sports areas. In this work, the integration of these areas is demonstrated, promoting a reflection about techniques and applications to collect, quantify and qualify some physical variables associated with the human body. These techniques are presented in various biomedical and sports applications, which cover areas related to diagnostics, rehabilitation, physical monitoring, and the development of performance in athletes, among others. Although some applications are described in only one of two fields of study (biomedicine and sports), it is very likely that the same application fits in both, with small peculiarities or adaptations. To illustrate the contemporaneity of applications, an analysis of specialized papers published in the last six years has been made. In this context, the main characteristic of this review is to present the largest quantity of relevant examples of sensor fusion and smart sensors focusing on their utilization and proposals, without deeply addressing one specific system or technique, to the detriment of the others.

## 1. Introduction

Given the growing demand for the development of intelligent monitoring systems, with a local processing or sensor network, this paper presents a review of the state of the art sensor fusion and smart sensors geared to sports and biomedical areas mainly during the last six years. In particular, it relates to how these technologies are present in several actions aimed at monitoring biological functions of individuals (biomedicine); exhibiting the use of biosignals for the execution of activities (biosignal interfaces); sports performance improvement of an individual (physical therapy and sports science) and recovery; and the correction of movements and ergonomics.

Taking into account the high amount of techniques on biomedical and sports applications, both sensor fusion and smart sensors are highlighted. In the literature, it is possible to find many related terms, such as: sensor fusion, multi-sensor, smart sensor, data fusion, smart devices, smart systems, fusion systems, among others. For a better understanding of this paper, smart sensors are defined as devices able to acquire, process and transmit/show data to users. On the other side, sensor fusion (which can be smart fusion or not) is a junction between two or more sensors present in the same system [1]. 

The sensor fusion concept is increasingly widespread and discussed these days, making it comparable to a Science [1]. Due to a large amount of features involved, it is unlikely that only one signal acquisition can provide a satisfactory compression system or variable analysis [2]. In general, sensor fusion is the combination of different data from sensors that may result in more complex analysis, which are not possible with the use of sensors singularly and/or separately [3]. In addition to data acquisition of different magnitudes, sensor fusion includes management and combination of this data with strategies to provide consistent and effective responses [1].

The development of fusion techniques is driven by the overview of a given system to be analyzed in order to improve the decision-making process into specific actions in the same system. The areas most affected by this technology are in commercial, social, biomedical, environmental, military, sociological, and psychological scopes of effects: in short, often interdisciplinary interaction [1,2,4].

When it comes to sensor fusion, there are two situations. During the first, the fusion is done on sensors with different signals [5]; while the second merges data, which is not necessarily of different magnitudes, but with equivalent sensors in different situations.

**Figure 1:**
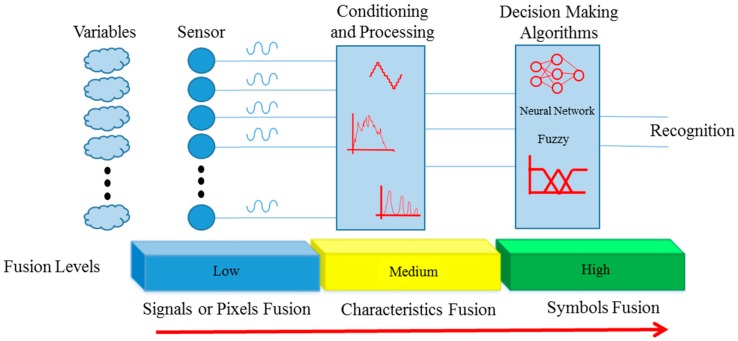
Summarized flowchart of a sensor fusion system.

Traditionally, its structure is composed of three levels, which act sequentially: acquisition and data merger, fusion of characteristics, and merger of decisions [1]. These three levels work with information in different classes, as shown in Figure 1. The first level (low) is composed of different sensors that collect signals from n variables, which can be physical quantities, chemical, biological or images (pixels). The second rating level (average) refers to handling and processing obtained signals, from which their main information is extracted. Finally, in the third level (high), there are manipulation classes, which create a fusion of symbols (characters, recognized information and strategies), and also where decision algorithms for recognition and transmission information are applied.

On another front, in cases where signal processing complexity is not so accurate, but there is the need for intercommunication between various points, there are smart sensors. Smart sensors are characterized by having their own communication system, which allows element sensor integration in a sensing network [6]. Unlike sensor fusion complexity, smart sensors are identified as having decision-making and communication present in a single system [7].

**Figure 2:**
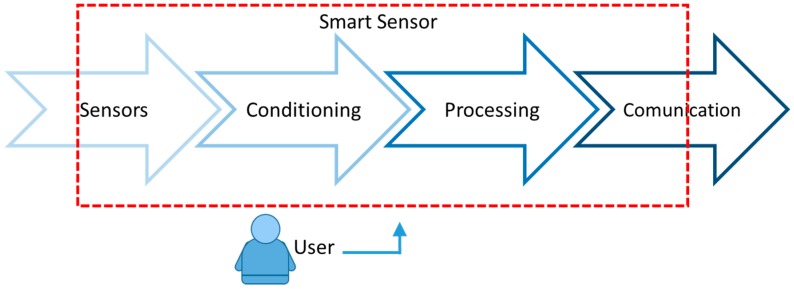
Summarized flowchart of a smart sensor.

In a simplified form, in a single module, there is all the acquisition of physical quantities by the sensor (s). These signals are electronically conditioned (by filters, A/D converters, etc.) and processed (by microcontrollers and/or microprocessors). Subsequently, the communication stage is responsible for data transmitting, using different means (by cable, wireless, Bluetooth^®^, Xbee^®^) in a network with other sensors for post processing elements and data analysis [7]. The user can configure the entire system remotely or on the device itself. This scheme is presented in Figure 2 [8,9].

The main applications of the smart sensors are: environmental monitoring, agriculture, transport and traffic, logistics, industrial, hospital, lighting, aerospace, energy conservation, automotive, and telecommunications [7,8,9,10]. Among these different areas, applications in health care and sports performance are also related with great emphasis [11,12].

Because of the similarities that exist between these technologies, as well as among the areas of analysis proposed in this review, this work is organized into three application classes: sports, postural and biomedical. 

Although it is not always clear how to discern between sensor fusion and smart sensors (and their related terms), the same occurs between biomedical and sports applications. However, it is necessary to make an individual classification for each case, taking into account operation and usage types. Therefore, this paper was not defined by the used technique, but by the function performed by those sensors in the main application. For example, it mentions smart sensors and sensor fusion applied in athletics (Section 2) and rehabilitation (Section 4).

The approach of this work has an extensive set of applications, and their technical description depends on the system’s development factors. Hence, the intention is to present the main idea of the sensor fusion and smart sensor utilization. Thereby, the technical specification of each system will not be shown, only the main concepts. The motivation for this different proposal is due to the large variety of devices used to achieve the same functionality, including those for commercial use and research purposes, besides including information unavailable to the public.

In the same direction, this review has avoided dealing with consolidated sensing techniques which rely only on the data fusion such as a motion capture system used in sports; but also chemistry biosensors, which deviate from the context and extent, usually having invasive applications which alone could give rise to a specific revision.

The aim of this paper is to demonstrate that there is a great interaction between biomedical and sports applications, especially in the case of the evolution of technology which has developed in both areas to quantify and qualify the physical variables that involve the human body. In such cases, there are variables that may be used for performance increase and for monitoring the health condition of a person (acceleration, heart rate, force, among others). This review is relevant in order to be able to enlace similar areas and cooperate with ongoing studies following the evolution of technology. 

In the field of sports, in Section 2, there are applications that aim to aid sports performance and to provide support for referees. As a connection, in Section 3, there are applications that work with principles that fit in both areas, sports and biomedical, especially regarding physical therapy. In the biomedical field, Section 4, there are applications with a rehabilitation purpose; general, physical therapy purposes; a monitoring purpose; and an aim to aid the diagnosis or execution of activities with the help of signals provided from the human body. A little contextualization is given to each branch, followed by some recent and relevant applications, and a brief analysis of the concepts of sensor fusion and smart sensors.

## 2. Sports 

Three types of data analyze the development of athletes in sports: physiological, physical and technical [13,14]. As physiological variables, the following can be highlighted: power (aerobic and anaerobic), lactate, glucose, oxygen consumption, and others [13,14,15]. Among physical variables, detach speed (average, critical, etc.), acceleration, and fatigue index, among others [13,14,15]. As examples of technical variables, there are starting time (athletics, swimming, etc.), proper execution of movements, repetitions of sequential movements, correct gait, posture during movement execution, among others [16,17]. The analyses of these variables corroborate with technical, physiologist and trainer assessments, used for decision-making and training implementation [13,14,16].

In sports, more and more applications have been developed as a result of data measurement, which is also a useful tool for performance evaluation. Information and analysis usually do not depend on the response of only one sensor but on the data fusion of multiple sensors, which must subsequently undergo processing and data communication in a dynamic and interactive way (preferably during exercise execution). However, in most cases post processing only occurs remotely [18,19].

The importance of instrumenting an athlete or accessory and analyzing real-time data is that it helps technicians and physiologists in assessing timely performance and the orientation of the athlete to develop it properly [20].

Sensor fusion, most commonly applied in sports, contains the following sensors: Accelerometer, Gyroscope and Magnetometer [12,18,19,21]. Keeping in mind that sensor fusion and smart sensor concepts can be used together, the selected applications in sports are presented and organized according to a specific sport or group of modalities, such as athletics, swimming, cycling, ball and puck sports and general applications.

### 2.1. Application in Athletics

Athletics is considered the motor base for other sports because it is difficult to find a high performance sport that does not require running, throwing or jumping [22].

There is a great demand for studies that enable the technician to conduct his/her analysis and guidance while in a training session, aiming for a higher growth rate of athletes’ performance [20,21]. In athletics, this convenience can develop a continuous interaction between coach and athlete, providing more useful guidance in a competitive environment and in the practice of sports, rather than in laboratories in further analyses [21].

In athletics, inertial sensors (accelerometer, gyroscope and magnetometer) are widely used, providing an evaluation of quantities, such as acceleration, angular velocity, and magnetic field, while providing orientation data analysis, as a quaternion and Euler angle [21]. The fusion of these data with a video signal can provide data that can justify the difference in performance between two or more athletes [21]. The work developed by [20] is an example of this analysis, in which it is possible to examine the inertial behavior of sensors according to the time displacement of two athletes [20]. With this information, the trainer can provide corrections, specific training and guidance to athletes in terms of starting time, positioning at starting time, among others.

In races, in addition to start monitoring, gait analysis is very important to the correct execution of movements during training and competition, since the coach will be able to monitor the biomechanics of this sport in real-time [23,24,25,26,27,28].

In order to analyze gait in sports, which depends on correct posture and movement, a system was developed using force and inertial sensors in an athlete’s shoe [29]. The application of three sensors, obtaining force, acceleration and angular velocity, comprises a sensor fusion used for the gait analysis of the athlete.

**Figure 3:**
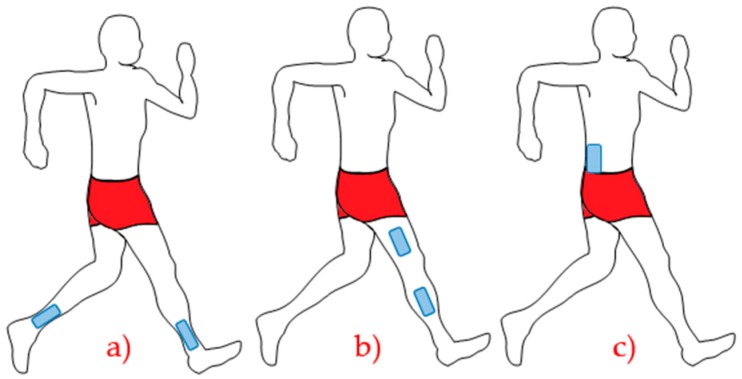
Examples of places to install smart sensors: (**a**) ankle; (**b**) thigh and tibia; and (**c**) lumbar.

Figure 3 illustrates some examples of places to install smart sensors according to their application. The places where sensors are installed will influence both results and the type of analysis to be performed, such as (a) installing a sensor on each ankle to analyze movements and range of movements; (b) installation of two sensors (thigh and tibia) to analyze the knee angle during gait execution; (c) a sensor installed in the lumbar can analyze hip movements, according to the pace [23,24,25,26,27,28,29,30].

The application of these concepts can be allocated to athletes for whom running is a bases of their sport (marathon runners, triathletes and sprinters). Athletics is not restricted to sports which are based solely on race, there are modalities using jumps (height, distance, triple, pole vault, etc.), accessories (hammer, stick, dart, disc, etc.), and obstacles (gap and barriers) [31].

### 2.2. Application in Swimming

Basic swimming movements can be divided into three phases. First, there is swimming style (freestyle, backstroke, breaststroke and butterfly). Second, there is the turn type, and third, there is swimming intensity (speed or resistance) [14,32,33,34,35,36,37,38,39,40,41,42,43].

Based on this information, sensor fusion and smart sensors may be applied in various forms in swimming, quantizing by numbers, graphics, and analyses. For example, to swimming type recognition; quantity of strokes and the time between them; and also swim and speed intensity [14].

Swimming requires two important measurements of variables to the development of the athlete, which are the resistance to the movement of the body in water and propulsion of the body in water, according to the efficiency of the arms during the movement [32,33,34,35,36,37,38,39,40,41,42,43,44].

To exemplify this, there is a system divided into two major blocks. One block is responsible for reading and storing data, and the other one is responsible for interacting with the data [44]. The function of the blocks is to perform inertial analysis of the limbs and the upper body during swimming, fusing these signals with images, allowing technical analysis of the type of swimming and its corrections through comparison of the swimming technique, according to numerical presentations (graphics), and video analyses [35,40,45].

Sensor fusion can be used for more specific goals, such as conciliating concepts of speed, power, and technique of a starting jump [40,45]. To illustrate this, there is a system that has the following smart sensors: sensors on the start platform, inertial sensors, pressure sensor on the edge of the pool, photoelectric sensors, and cameras and sound signals [40,45].

In this system it is possible to detect the reaction time of the athlete to the starting signal, the power generated from jumping off the platform, his/her movement and technique, jump time (flying), among others [40,45].

However, it is not only the monitoring of athletes that can be applied to sensor fusion. Smart sensors and their fusion are also applied to the automation of sports and information provided to scouts. In recent years, the number of systems that update the data of athletes (number of strokes, and the time between them) in real time has increased, evaluating several athletes simultaneously, as well as systems that help conduct training for several swimmers [39,43,46]. These systems have been developed so that technicians would only be concerned with the movements of athletes and not the sequences of the activities or taking notes and data during training [39,43].

### 2.3. Application in Cycling

In cycling, studies are applied to both the performance improvements of riders and for the rehabilitation of injured athletes, preventing future injuries [13,19,47,48,49,50,51]. Different types of data are used, such as power, oxygen consumption, heart rate, effective force to the pedal, and biomechanical measurement of the variables (such as foot and knee angles during exercise, correlating them with cadence) [13,19,47,48,49,50], which are studied considering their relevance in athletic performance [19].

**Figure 4:**
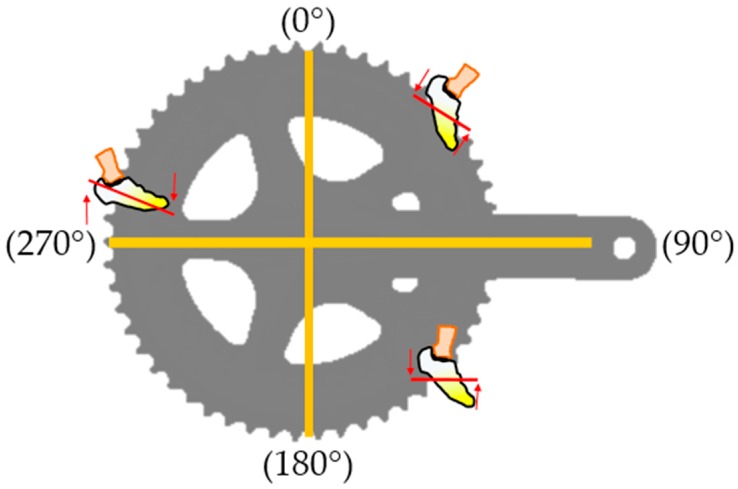
Power application phases and foot angle correction on the pedal while pedaling.

Some analyses can be performed in real time so that corrections can be made during exercise practice, thus providing pedal stroke profiles of the athletes. The foot angle of the athlete on the pedal is crucial to fully transfer the strength of the foot to the pedal [19]. To visualize the correct foot position (angle), a smart sensor was developed that analyzes the angle of the foot by inertial sensors, and presents the correction in an application on a mobile device, so the rider can reposition their foot [19] (Figure 4).

The crankset, shown in Figure 4, along with the pedal, rotates 360° during exercise execution, allowing the analysis of the cycling in two phases. The first, which starts at 45° from the top (0°), generates power to move the bike, and the second phase, which happens when the set reaches 135° from the top, starts the recovery process, in which there is no motion generation. The two assessments previously mentioned are displayed in Figure 4.

When power is applied outside the region of movement, pedaling efficiency decreases, which may lead to a future injury [15]. Various techniques have been applied to measure the pedaling angle, but the use of accelerometers is one of the most efficient and the signal obtained has less noise. Once data is acquired, the signal is processed, which evaluates if the feet position is the most suitable for the best performance of the rider while pedaling, and helps to prevent injuries.

In addition to pedaling technique, there are other key measurements for cycling, such as quantifying the physical condition of an athlete. As a physiological variable to be measured in cycling, power output stands out for being measured during training, competitions, and even in laboratory evaluations [13,47]. The power meter used in cycling is a device that has a fusion of two sensors (force and speed) and transmits data via wireless communication, being characterized as a smart sensor [13,47,48,49,50,51].

The speed sensor is commonly found in two different forms. The first, and most common, is a magnetic sensor installed on the crankset, detecting the passage through a fixed magnet installed on the chain stay. The second form is presented in Microsystem Electromechanical (MEMS) form, being the gyroscope responsible for measuring the angular velocity of the pedal [47].

The power or torque sensor is responsible for measuring the deformation in a mechanical part of the bicycle, and this part can be the pedal, the crankset, the chain ring, the rear hub, or even the chain stay [51]. This sensor is called Extensometer, which transforms the intensity of the mechanical deformation into electrical resistance changes [47].

After torque and speed data fusion, the power values in real time are displayed for the rider. A device called Head Unit, which is placed on the bicycle handlebars, performs the interface with the cyclist [13,47,48]. With the evolution of smartphones and mobile devices, integration with power meters is facilitated by two wireless communication protocols: ANT^+^ and Bluetooth [13,48].

Having such examples of sensor fusion applied to cycling, it is clear that the analysis can be of both the techniques and physical condition of the athletes, helping the sport as a whole. Cycling is a sport that has endurance (road, hour record, etc.) and speed (time trial, track, etc.) racing categories, with the applicability of the concepts of sensor fusion and smart sensors being vital to all of them [13,47,48,49,50,51].

### 2.4. Ball and Puck Sports

#### 2.4.1. Applications in Football (Soccer) 

Soccer, in particular, is a very susceptible sport for refereeing errors with constant slip ups regarding offside decisions and even goal validations [52], which directly influences the course of the match and the final result.

Goalpost instrumentation, using sensor fusion, has recently been proposed [52,53,54] based on two techniques to scan a certain area. The first is based on cameras installed in the stadium structure, making a decision according to the position of the ball related to the goal line based on the image of three different cameras (at least) at the same time [53]. The second technique is based on magnetic field sensors installed in the three goalposts, where the decision would be made based on the magnetic field change [54,55]. Both techniques process signals from sensors and transmit them to the referees by wireless encrypted communication [53]. These systems can be applied in other sports besides soccer, such as hockey (ice and grass), basketball and water polo [54].

While the first technique is based on image fusion, the second also uses smart instrumentation of the ball, which is loaded with a passive electronic circuit and the goalposts present a low frequency magnetic field generated by the system. Any variation in the magnetic field behind the goal line is detected and automatically confirms, or not, the passage of the ball [55].

The importance of smart sensors and sensor fusion in ball sports goes beyond monitoring rules and objectives, also being applied in the physiological measurement of variables to evaluate the physical performance of each athlete [55].

**Figure 5:**
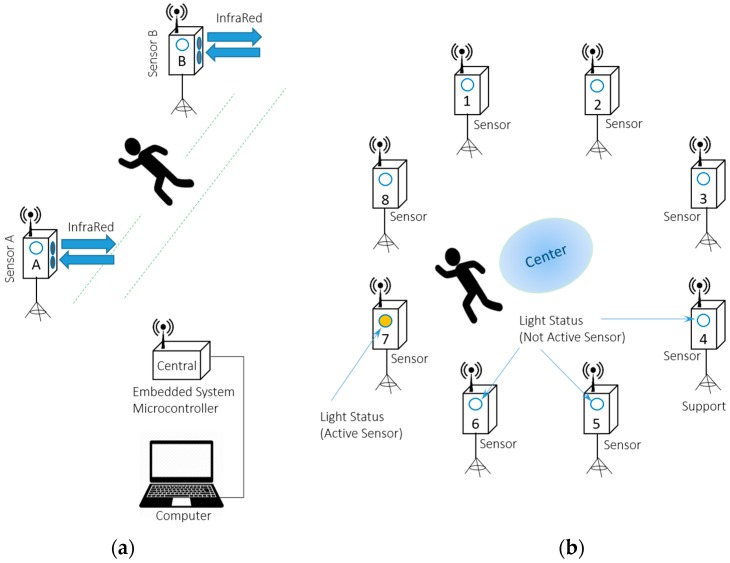
A system for collecting collection time in continuous running (**a**) and agility tests (**b**).

Regarding physical evaluations, running evaluation systems can be exemplified with sensors and timers, which in parallel with physical examination sensors (fatigue, heart rate, etc.) are transmitted to a signal processing center, as exemplified by Figure 5 [56]. The system displays the running time between the towers. With this given time and the distance between towers, it is possible to calculate, along with other parameters, physiological power, fatigue index, among others [15]. Figure 5a exhibits a smart sensor topology for collecting time in continuous running between infrared or optical sensors (A and B), and it can be sprints or even laps. Additionally, the system shown in Figure 5b is used for agility tests, composed by many sensor towers (is showed 8 towers), where the measured value is the time taken for the athlete to trigger the active sensor and return to the center, which is indicated by lighting the tower to be triggered. Both systems has a central tower with a embedded system microcontrolled to collect data, store them and also enable them to transfer to a computer.

To survey the physical performance of an athlete in the field, a GPS (Global Positioning System) was used in each player to collect data such as speed, position, acceleration, time of each activity type, among others [57]. Thus, sensor fusion use for soccer analysis is important for rule application, and for the monitoring and assessment of players.

#### 2.4.2. Applications in Basketball

The application of smart sensors in basketball includes real-time analysis of passes, shots (jumps), drives, and dribbles [58,59] in both games and practices [58,60,61].

To illustrate the individual analyses, the ball instrumentation stands out, performing data collection to compare shot types; ball output angle from the hand of the athlete; the angle at which the ball enters the hoop; and speed and flight time of the ball [61].

In [61], the proposed basketball instrumentation was developed using nine accelerometers installed on the ball, which communicate with mobile devices, allowing the user to retrieve data through an application installed on the device. This type of analysis helps corrections of movement and shots in order to improve quality and accuracy of the field goals [61]. As for movement and intensity analysis inside the court, smart sensors are installed on the body of the athlete, creating fusions due to the physical performance of the athlete on the court [58,59,60].

These fusions are usually based on smart sensors installed on the body of the athlete, such as GPS, instrumented insoles, inertial sensors, and cameras [58,59,60,61]. Together, these data are analyzed according to the performance of the athlete, generating reports and feedback to coaches, physical trainers, physiologists, and physicians.

#### 2.4.3. Applications in Sports with Protective Equipment

Within team sports, there are modalities that require protection to the athlete because of the impacts and intensity that the game offers, such as hockey and football. In these cases, smart sensors and sensor fusion application enable the analysis of the impacts suffered by the athlete [62] which also helps to provide knowledge to the development of protective gear. Several recent articles in the area show the concern about monitoring possible concussions and other injuries to the head that may be caused by an impact, especially in ice hockey and football [62,63,64,65,66,67,68,69,70].

**Figure 6:**
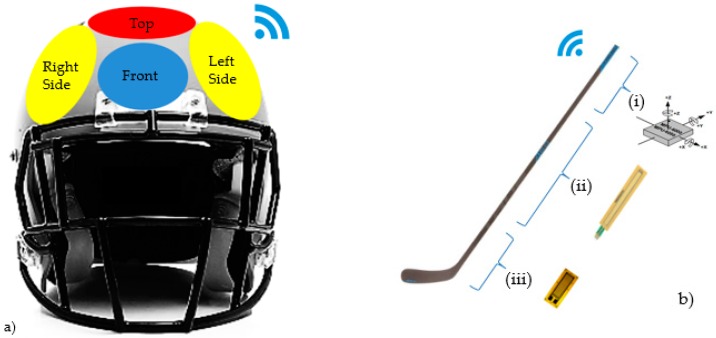
Instrumented smart helmet (**a**) and instrumented smart stick (**b**).

Most of the impact monitoring systems in the game are composed of smart inertial sensors that can transmit the following values: impact acceleration, impact time, impact local (head or part of the body), impact direction, and the amount of impacts in sequence (if more than one) [63,64,65,66,67,68,69,70]. As the largest target of the studies are impacts on the head, the most instrumented protective equipment is the helmet (Figure 6a), since its approach has gained more relevance in the light of concussion cases in these sports [64,67,69]. However, the vest can also be instrumented [71].

In addition to safety equipment, in hockey there is also the fusion of smart sensors on the stick, which analyze the movements of the athletes with the stick, the force of the strike, and the position of the hands on the stick [72]. The fusion of three sensors installed on the stick can be used (Figure 6b): inertial sensors at the top of the stick to analyze the movements of the stick in the hands of the athlete (i region); linear potentiometers to identify the hand position on the stick of the athlete (ii region); and lastly, strain gauge to analyze the deflection of the stick at the time of the strike (iii region), thus monitoring the force at which the puck is released [72].

### 2.5. General Applications

In sports, heart rate is correlated to the effort of the athlete; its comparison with its thresholds provides performance and fatigue levels in physical activity [73]. For this measurement, [73] which can be used in volleyball, where heart rate sensors are installed on each athlete, monitoring in real time and transmitting to the coaching staff. However, this measurement type can be used in conjunction with other sensors, executing data fusion in other sports such as athletics (track), cycling, swimming, soccer, and basketball [13,48,73].

In addition to the already mentioned types of movements, other types of movements are essential in sports, such as swinging, which is characterized as a complete movement with a bat or racket to have contact with the ball at the optimum time and angle, which is used in sports such as tennis, golf, baseball, cricket, among others [74]. Based on this movement type, a system was developed to analyze the swinging applied to golf. Inertial sensors are installed on the golf club, graphically raising the trajectory of the bat, as well as the position and time until contact with the ball. These analyses can lead to a breakthrough in golf teaching and also provide feedback to coach and athlete during training and competitions [74].

The use of the concepts of sensor fusion and smart sensors can be applied to various sports and types of analyses, as presented in Table 1, which displays the sports and the type of analysis.

## 3. Applications between Sports and Biomedical Areas

After the presentation of the application of smart sensors and sensor fusion in sports, this topic reports the applications that can be used for sports and biomedical tests, all with the same equipment. The objective of this topic is to introduce a connection between sports (Section 2) and biomedical applications (Section 4). Three topics were selected to introduce smart sensors and fusion sensors to both areas: plantar pressure, electrical activity of muscles, and ergonomics.

### 3.1. Plantar Pressure

**Figure 7:**
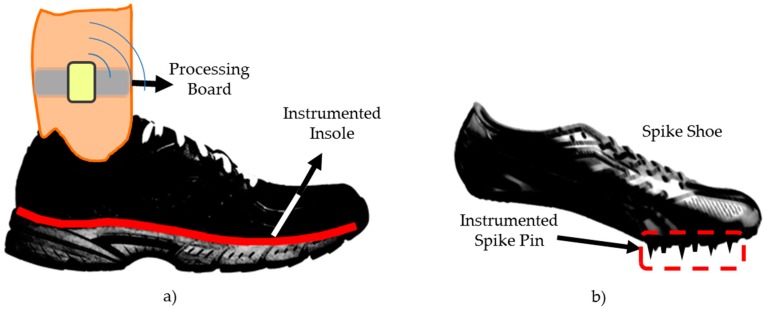
Plantar pressure smart sensors: (**a**) in-shoe insole and (**b**) spike shoes for sprinters.

To perform plantar pressure measurement in dynamic situations, the best system currently displayed is called in-shoe, where a plantar pressure acquisition system is installed inside the sneaker or the footwear of the individual [86,87]. This system model is based on measuring the plantar pressure between the foot of the individual and the outsole of the footwear, having an interface between the parts of an instrumented insole [17,86,87,88,89], as featured in Figure 7a.

According to [86], in-shoe systems need some basic requirements for safe operation, such as being mobile, having the least number of cables, being comfortable inside the sneakers, lightweight (about 300 g), low cost, and low power consumption. This system is characterized as a smart sensor because it has acquisition, processing and wireless transmission of plantar pressure data [86,87].

In addition to commercial systems, other in-shoe systems are developed to research and specific studies, such as an instrumented insole that was developed to plantar pressure measurement in heavy human activities [87]. As the main application, this insole has been applied to a landing simulation parachute, which has a high impact on feet in contact with the ground [87]. This system uses eight sensors that present an output voltage according to the internal material resistance that changes when a mechanical force is applied to it. The system is microcontrolled and transmits data by wireless communication modules [82].

For deeper or more specific analysis, there are other methods, such as the distance between feet, gravity center, pressure percentage by foot, etc. [17,86,87,88,89]. These plantar pressure concepts can be applied in high performance sports, not only in training or in the analysis of the assessments, but also, for example, in track sports, snowboarding and soccer [16,24,79]. To achieve this, miniaturized sensors were developed and installed within the pins of the athletic spike shoes (cleats) [16]. The instrumented spike shoe contains six pins with sensors installed in each of them, as shown in Figure 7b.

### 3.2. Muscle Activity

Muscle behavior analysis during a physical activity can be performed using Surface Electromyography (sEMG) [90,91,92]. In sports like weightlifting and powerlifting, there are movements of lifting metal bars with weights attached to their ends [91]. 

Weightlifting activity can be harmful to the body if not performed correctly and requires continuous assessments to avoid injuries during training and competitions, such as in Olympics and Paralympics [92]. To illustrate sEMG use, which can be applied in both sports and biomedical applications, there is a protocol for assessing muscles of the trunk activation when a weight is lifted from a resting state [90]. The intersection of sEMG values with video imaging analysis can generate a data fusion to perform the comparison of muscle electrical activation with body position in weightlifting.

The movement of lifting a weight from the floor may occur, even at low intensity, in everyday and domestic activities. These activities are responsible for most of the injuries that occur in the back, particularly in the lumbar region. A data fusion such as this allows the characterization and quantization of the muscles of the trunk activation, being baseline studies for the injuries and rehabilitation of athletes and people deprived of some movements [90].

### 3.3. Posture and Ergonomics

Several systems are able to conduct a posture analysis, which can operate in improving sports performance and in recovery and rehabilitation systems for medicinal purposes [93]. For example, heart rate contribution with trunk inclination and acceleration data allows a smart sensor to perform the measurement of movements and positions for the physical classification of postural activities, especially in the detection of abnormal conditions susceptible to an emergency [94].

In most cases, sensor fusion and smart sensors of those applications are developed by means of an inertial sensor. Accelerometers, gyroscopes, and magnetometers can be used for posture monitoring and for some ambulatory functions by extraction, via Kalman filter, of the orientation of a person [95]. In therapeutic applications, inertial sensors are used to measure posture inclination angles, which allow a system to perform feedback with vibrotactile stimulations to people in the rehabilitation process [96]. However, there are exceptions, such as a smart sensor developed with inductive sensors sewn into a T-shirt that permits the analysis of the spine curvature [97].

It is also possible to notice that the inertial sensor can be associated with other sensors for recognizing and tracking functions and daily movements. For day-to-day activity recognition, the fusion accelerometers and radio frequency identification sensors (RFID) correlate both movements and the amount of calories burned by the exchange of gases, present in different activities [98]. The fusion of inertial sensors, a belt of one sonar sensor and ultrasonic sensors inserted in shoes, are used to estimate the posture of the lower limbs in real time, with obstacle detection in unknown environments [99]. As for outpatient movements, smart fabrics (e-textiles) and smart sensors are used for an angular measurement system (goniometer) of the knee joint, with better performance and less errors than commercial systems [100].

However, regarding posture analysis, sensor fusion and smart sensors can assist in the rehabilitation and in the treatment of diseases. In clinical systems, a combination of accelerometers and gyroscopes can help to measure balance, mobility, and movements, such as standing up, walking, turning, and sitting again—especially for people with Parkinson’s, who may be monitored by a smart sensor [101]. For monitoring people with a neurological disorder and chronic diseases, data fusion of acceleration, angular speed (gyroscope), and video images are used to assess postural changes [102].

In ergonomic systems, a stress and sleep quality estimator were developed using Electrocardiography (ECG) in the form of a smart sensor [103]. Another example is a fusion wearable system, based on a piezoelectric sensor array with tri-axial accelerometers, which are sewn onto lycra clothing. This system is used to analyze spine curvature and lumbar spine bow [104].

After the presentation of applications that can be directed as an intermediate for sports and biomedical applications, Section 4 will present smart sensors and sensor fusion applications, besides data in biomedical engineering and its specifics.

## 4. Biomedical Applications

The sensor fusion concept is widely used in biomedical engineering. Among its applications are some simple cases, such as ECG use, Arterial Blood Pressure, and Photoplethysmography (PPG) for monitoring cardiac signals. Even if some techniques have their advantages when used separately, together they provide greater robustness and reliability for data analysis in diagnosis [105]. However, they may be applied to systems with deeper analysis, such as brain function understanding. Due to the complexity of the brain, which requires more refined and detailed function mapping, the adoption of only one technique presents difficulties. Using space-time signals obtained by encephalography (EEG) and magnetoencephalography (MEG), adding functional magnetic resonance imaging (fMRI) enables the analysis of how different parts of the brain contribute to activities related to perception and cognition [106]. To achieve this goal, three different techniques, with different sensors and signals, are used together.

On the other hand, there are situations where diseases cannot be diagnosed by simple methods, such as potentially malignant tumors, due to factors such as low sensitivity, high-risk of a false positive, and a limited number of spatial samples (occasionally in biopsies). Fused sensor information helps the doctor make a more accurate diagnosis [106], which is facilitated by the development of miniaturized electronics and wearable systems [106,107].

There are applications that use data matrices to acquire signals. However, they are composed of the same data type. Biopotential collection systems, such as electromyography (EMG) [108], EEG and ECG, or anything that uses the same type of sensor [109] should be handled with care because more than one electrode can be used to capture the signal. If the type of the signal obtained is the same, the system is classified as a multisensor system. Its nature classifies it as sensor data fusion; an example of this is the use of an array of electrodes to collect signals from surface EMG in pregnant women to monitor uterine contractions [110]. Thus, this work contributes to promote a multiple data analysis in a non-invasive way.

In parallel with data fusion and sensor technologies, smart sensors have also been widely used in biomedical applications to acquire and process data to be used in assisting with diagnosis**,** self-diagnosis [111], telemedicine [112], home monitoring (home care) [113], and to save lives [114]. In the construction of these sensors, some electronics principles and/or physicochemical reactions, such as biosensors, are noticeable. These biosensors facilitate the development of smart sensors because they can be miniaturized and implanted. Some present themselves with the concept of MEMS, which are used for various applications such as treating tumors, controlling blood glucose levels, and releasing therapeutic agents in response to biomolecular and physical stimuli to minimize medical care personnel intervention [115].

There are numerous examples of smart sensors, such as the m-Health (mobile-Health), a simple wearable device that monitors cardiac activity in real-time [116]. Meanwhile, more complex systems require the use of smart sensors, such as a prosthesis which assists people with degenerative retinal diseases—this is still being tested [117]. This system uses a camera to capture the signals and an array of electrodes to stimulate the eye, reinforcing the image. Finally, one of the greatest conveniences of smart sensors is their ease of replication, which allows them to be commercially developed [118], such as an EMG monitoring system [119].

Next, equipment, devices, and systems that use sensor fusion techniques and smart sensors through the main biomedical applications, will be covered. Though there may be some overlap, the examples were separated into classes: patient monitoring in a hospital/clinical environment, rehabilitation, home monitoring, self-diagnosis; and other relevant applications that do not fit in the above.

### 4.1. Patients Monitoring in a Hospital/Clinical Environment

In the development of systems for patient monitoring, sensor fusion techniques can be used for the analysis of a more complete and general condition of the patient. In a hospital environment, the use of a single system to insert sensors on the patient, such as the body temperature, heart rate, ECG, breathing rate, and acceleration of the body, is common [120]. These systems may be wearable [121]. These data are sent to a central processing unit, and algorithms identify the behavior of an individual [120], verifying, for example, if he/she fell down or if their physical condition is not stable. In integrated systems, a robot can be sent to meet the patient, which recognizes him/her through a camera and a 3D spatial analysis’ LED (Light Emitting Diode) [120].

The technique of fusing data from multiple sensors is exemplified in an endoscopy system with eight inertial sensors inserted into the endoscopic tube, determining its position and location [122]. The use of multiple sensors provides complete guidance to the doctor in only one device, and therefore, results in a better correlation of the orientation with collected images from the tube, offering control of movements and lessening the chance of internal organ damage [122].

Image data can also be fused, such as Positron Emission Tomography (PET) with ultrasound images, which provides reliable clinical data to be presented in real time, using computer tomography scanners [123]. On the other hand, data fusion to combine electromagnetic navigation with imaging systems allows the performance of a biopsy of small lesions with high accuracy, which can subsequently be used in clinical environments [124]. In exam and surgical systems (intraoperative), the obtained data of endomicroscopy fused with an ultrasound signal allows complementary information to the execution of transanal endoscopy microsurgeries [125].

**Figure 8:**
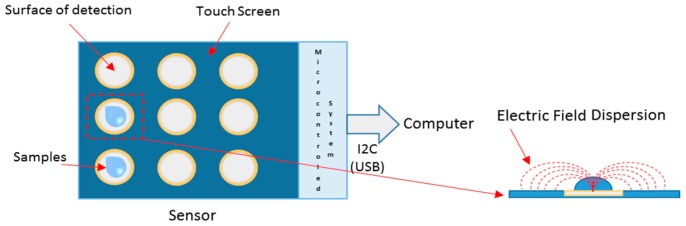
A detection system for a urinary infection.

For clinical applications, an example of a smart sensor is based on a planar capacitive sensor, used for the measurement of urinary tract infections, to decrease the time of exams [126]. Instead of a laboratory analysis, a capacitive sensor was used to detect the concentration of the *Escherichia coli* bacteria in urine samples, since bacteria alter dielectric properties of infected material. A capacitive plate, containing a touch screen, with nine deposition samples areas, was proposed as suggested in Figure 8. Inserting the sample, the electric field dispersion is characterized according to the properties of the sample. Sensors are coupled to a microcontroller that collects the signals, treats them, and sends the data to a computer [126].

Another example is a system of a smart sensor that was introduced to monitor the glucose of the user [127]. Thus, data is sent to a communication system with insulin pumps, and the feedback signals the release of medications into the patient. A commercial sensor system is coupled with modules containing software tools, and together they generate alerts for a low and high concentration of glucose.

A set of capacitive sensors is used for clinical monitoring with a sensor for the evaluation of urinary continence. The set contains an array of capacitive sensors enclosed in a single system (smart), inserted into the urethra to measure its pressure [128]. Capacitive sensors were chosen because the application requires flexibility for insertion into the human body, in addition to the reliability of the measurement of liquids in an in vivo environment. The presented set of sensors includes nine elements, arranged in a single row, providing data to be analyzed together, which is a differential as the authors present.

**Figure 9:**
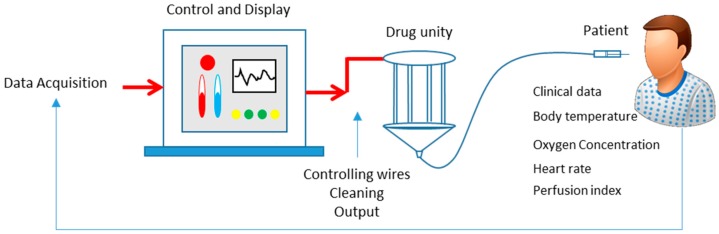
A monitoring system and overview of medication control.

Also in the field of smart sensors, there are systems that combine different signal acquisitions to the same application with combined treatment, making a data fusion. This is the case of the continuous patient monitoring system, suggested in Figure 9, which gathers body temperature (thermometer), perfusion index, oxygen concentration, heart rate (these latter three coming from a pulse oximeter), and other clinical data (respiratory rate and urinary concentration) [129]. This system can be controlled remotely by a physician and acts by controlling the dosage of the medication and frequency for a particular patient.

With the ascent of wearable devices, the union of smart sensors and sensor fusion has become increasingly present, such as the integration of ECG electrodes, a microphone, a pulse oximeter, an accelerometer, two respiration bands (thorax and abdominal), a humidity sensor, a room thermometer, and a body thermometer in a T-shirt [130]. All these sensors are integrated**,** providing a wearable system for monitoring chronic diseases. The system is also characterized as a smart sensor as the signals are jointly processed in a central position, and later there is communication to a recording system.

### 4.2. Rehabilitation

Rehabilitation system devices intend to help people who have suffered accidents, disease or infirmity, in order to recover and restore their condition (physical, sensory, and mental) [131]. As an example in this context, [132] presented in 2012, a rehabilitation system for people who have suffered strokes to perform daily exercises using a vision system with the fusion of inertial sensors. The inertial sensors are inserted into utensils (spoon, fork, and cup), allowing the analysis of position and vibration, while the vision system enables the distinction between healthy and paralyzed areas of the body, in order to generate a report about the health of an individual.

Electromyography and motion sensors are an example of sensors fusion and have been widely used for rehabilitation systems. EMG signals (from electrodes) and motion sensors in three dimensions can correlate muscular effort and spatial position to evaluate the muscle recovery process [133]. A vision system and multiple fusions of EMG channels exhibit improvements in the analysis of the displacement of people walking, with a focus on people suffering from neuromuscular complications, such as strokes and cerebral palsy [134].

A smart sensor using EMG signals, inertial sensors, and a flexible polymer sensor helps people during the rehabilitation process of the knee [135]. The addition of the latter, compared with inertial sensors, provides a better joint analysis by its flexibility and passive electrical nature.

The development of prostheses and exoskeletons, in robotic control settings, has also used EMG signals associated with other sensors [136], such as in [137,138,139,140]. In [137], an application is presented where EMG electrodes are attached to the arm, inertial sensors are positioned in a prosthesis, and vision systems (allocated in the head) are used for the movement of a prosthesis more efficiently than only using EMG. In [139], a combination of inertial sensors and EMG in the arm is used in conjunction with a virtual simulation device for the restoration of the functions of the upper limb. Other systems invest in uniting EEG with EMG and inertial sensors, monitoring and suppressing involuntary shaking of the body [138]. There are methodologies, such as tracking [140] which, with inertial sensors, map EMG space traversed by the arm to be reproduced by a robot, which can be associated with a prosthesis. This methodology can efficiently overcome nonlinearities that exist between the EMG signals and the position of the limbs.

**Figure 10:**
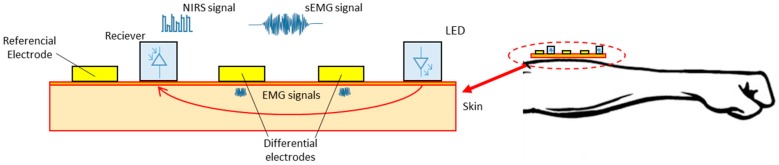
Fusion system of signals from NIRS (Near-Infrared Spectroscopy) and EMG (Electromyography).

In a different study, there is an EMG fusion with near-infrared spectroscopy (NIRS), which uses three EMG electrodes for EMG capturing and a transmitter-receiver pair (LED and Photoreceiver) to spectroscopic signal, as indicated in Figure 10. These two combinations of sensors facilitate the understanding of muscle activity (electrophysiological and metabolic) and assist in the development of prostheses [141].

To prevent leg weakness or atrophy of the nervous system, an instrumented cyclic wheelchair (with pedals for locomotion) is proposed as a rehabilitation option [142]. Sensors fusion occurs with angular position encoders, an inertial sensor, a potentiometer, and a laser. The angular velocity data of the wheel of the chair (encoder) and of the whole chair angular velocity (inertial sensor) allow the wheelchair dynamics to be monitored, while the torque on the pedal is measured (potentiometer). It also ensures that there is no object obstructing the path (by the laser). This data promotes the engines to increase the power of movement, enabling atrophied muscles to increase their strength, as well as their correction and recovery.

**Figure 11:**
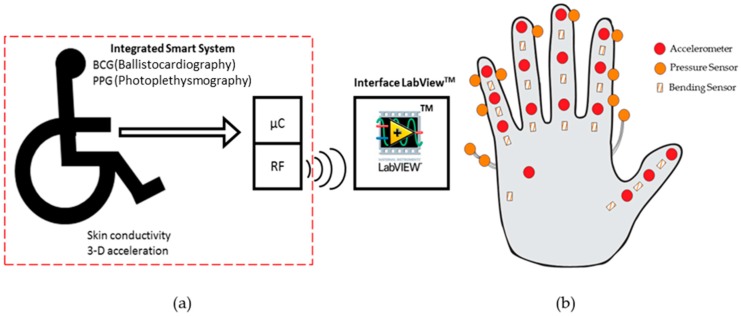
(**a**) Monitoring of vital signals via wheelchair and (**b**) Instrumented glove for assessments of rheumatoid arthritis

Cardiopulmonary evaluation (obtained by fusing photoplethysmography-PPG sensors, skin conductivity, and ballistocardiography-BCG) and motor activity (with a 3-axis accelerometer) enable the status of a wheelchair to be monitored [143] as featured in Figure 11a. The techniques used provide physiological discrete parameters, such as respiratory and heart rates. The continuous monitoring of these parameters allows for both a shortened hospitalization time and assists in monitoring rehabilitation. A microcontroller (μc) digitally processes all signals of the sensors and data are transmitted to a computer or mobile device. A program is proposed for the application of procedures and the analysis of collected data, providing further evaluation and/or diagnosis [143].

Smart sensors can also support data fusion, and this is exemplified in the instrumentation of a pair of gloves (illustrated in Figure 11b) for monitoring rehabilitation progress of patients suffering from rheumatoid arthritis at the wrist. Bending sensors, accelerometers, and force sensors are used for measuring the angle of the joint of the hand and fingers [144]. Bending sensors measure flexion of fingers and wrist rotation; force sensors measure the interaction of forces by the Kapandji index (graduating movements of the big toe related to other hand regions); and the accelerometers, located on the phalanges of the fingers, assist in reading the position of the fingers. Data fusion and respective analysis are performed in microcontrollers, which brings dynamic, rather than static, analysis, as goniometers used for similar applications are static.

To assist people with the rehabilitation of Parkinson’s disease, load control, and speed sensors can be used to instrument a bicycle, while its user can use sensors to monitor heart rate [145]. Storage systems and data transmission centralize the influx of signals and allow the data merger to parameterize the most appropriate exercises for the individual in question. For patients with Parkinson’s, it is proven that high pace and high intensity exercises improve their motor functions, which are made possible by the equipment. The proposed equipment, in the form of a smart sensor, enables improvement of motor function, monitoring the condition at the same time as it is modifying the situation of the exercises.

Smart and fusion concepts are found in rehabilitation systems as well as in auxiliary equipment and for the support in these systems. A set of accelerometers (analog and digital) and gyroscope (digital) enables one to retrieve information about any abnormal or dangerous situation on the device during its operation (rehabilitation process of a patient) [146].

### 4.3. Monitoring and Diagnostics Aid

To provide assistance with diagnostics and monitoring systems, there are tools that combine various technologies which infer parameters that may indicate a particular behavior or abnormality [147]. Examples of this are the smart sensor in [148] (inertial sensors to detect falls and night epileptic seizures) and in [149] (proposed for measuring heart and temperature rates). Most of these systems are developed for patient monitoring outside the hospital (homecare) [150], based on the cost savings that medical equipment adds to the treatment [151]. Furthermore, systems working with sensor fusion and smart sensors techniques mostly can be used remotely, relying on wireless communication modules for data analysis on other devices, using biotelemetry [152] and e-Health concepts [153].

Photoplethysmography (PPG) techniques, temperature measurement, and the use of acceleration to monitor heart rate, body temperature, falls and inclination of a patient while sleeping, in a smart sensor [150] is an example of these mentioned systems. Similar to this is the remote body temperature measurement by thermoresistive sensor and heart rate by PPG, which can detect hyperthermia, hypothermia, tachycardia, and bradycardia [111]. In [151], a smart, simultaneous sensor with a single chip containing physiological date for temperature, glucose, protein concentration, and pH (hydrogen potential), obtained by resistance change, measuring tension, flow, and capacitance. A biotelemetry system is proposed in [152] for arterial blood pressure measurement (with a MEMS sensor based on piezoresistive principles) and body temperature in a smart sensor, which aims to monitor hypertensive patients.

Graphics platforms used for the acquisition, processing, analysis, and preparation of data, such as LabVIEW^TM^, are one of the solutions found in monitoring systems. Presentation, processing and analysis of signals, obtained from an ECG acquisition smart sensor and processed in a Digital Signal Processor (DSP), were developed with LabVIEW^TM^ [112]. Data is transmitted to a computer containing such a platform. Also with wireless transmission and LabVIEW^TM^ use for data analysis and remote monitoring [154], a smart sensor with the ability to measure sodium, potassium, chlorine, and pH was developed to observe electrolyte levels. In both cases, it is perceived the smart sensors with a monitoring function combine data acquisition platforms, such as LabVIEW^TM^. These techniques allow for monitoring to be done at home with computer software, since the nature of these processes in clinics demands large equipment and laboratory analysis.

**Figure 12:**
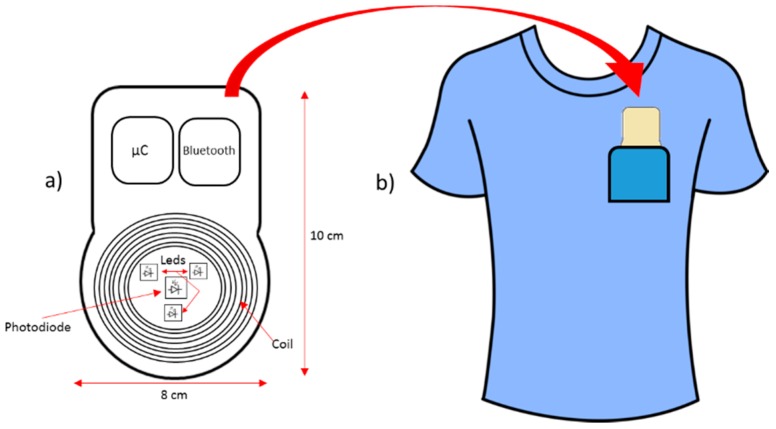
Model of a system for measuring lung volume through an inductive sensor and PPG, featured (**a**) its circuit and (**b**) its size to be placed in the pocket.

Wearable sensor fusion and smart sensors are solutions for monitoring patients, like for example, the fusion of PPG reflective (commonly used to measure and record volume changes in a portion of the body or organ) with magnetic induction sensors. Both techniques enable cardiorespiratory monitoring without using elements that come into contact with the body of the patient [155]. A Bluetooth module consists of a coil made of printed circuit tracks, and a photoplethysmography optical sensor (composed of LEDs) processed by a microcontroller. The device is designed in a way that it can fit in the front pocket of the shirt of the patient, as shown the model in Figure 12.

Another example of sensor fusion in wearable systems uses the respiratory rate information (obtained by accelerometers) and ECG (acquired by electrodes) deployed in a T-shirt [156], which is optimized for monitoring in neonates [157]. Data provides vital information for parents of newborns, who monitor them on a remote system that keeps the accelerometer, inserting a gyroscope, and changing the ECG sensor to a capacitive sensor that does not need contact. In [158] a smart sensor is displayed. Performing fusion of the three acquisitions simultaneously allows the monitoring of the heart, breath, and movements. Other examples are EMG textile electrodes, piezoresistive sensors, and inertial sensors, which are fused in a wearable system in shoes, trousers, shirts and gloves, or instrumented to assess the patient in stroke treatment [159].

Many other wearable examples use inertial sensors, which are frequent in applications aimed at monitoring. Accelerometers and gyroscopes can detect and monitor balance of the body, revealing Alzheimer’s evidence in its initial state by merging this data [160]. The data fusion from multiple smart sensors, based on accelerometers, can assist in the evaluation of diseases that affect vascular and neurological systems by exposing the individual to vibrations coming from occupational equipment [161]. Data are recorded and remotely merged into microcontrollers or DSPs to estimate the average amount of daily noise that a worker is exposed to. When they go beyond the safe limits, an audible alarm is triggered, and a message to the operator to stop using the machine appears.

The EMG monitoring area has advanced in electrodes’ combination for smart sensors, as in a device that uses EMG signals from the stomach, which records signals from the activity of the stomach noninvasively [114,162]. Moreover, for monitoring kinesiological functions, neuromuscular diseases, and motor activity disorders, [163] a smart sensor is presented with electrodes constructed on a printed circuit board. In both cases, there are processing and data transmission systems.

In diabetes management, a major problem is in the determination of the quantity of glucose in the blood, which has good accuracy only by clinical examination. The development of less invasive and commercial techniques, such as optical gauges [164] have tolerance results within ±20%. More crossing of techniques can reduce the error range, providing greater reliability to the patient in this high demand area [165,166]. This is the case for data fusion of capacitive and optical sensors [167]. Optical spectroscopy and dielectric measurement enable the estimation of the level of glucose through the skin, proven by the same study [167], which resulted in high accuracy. Another case of fusion occurs with ECG and pulse oximetry techniques in monitoring diabetes [168] by correlating this disease with heart problems. Both measurements are made in separate parts of the body. However, the proposal links them in one area as the functionality of a low-cost smart sensor.

Under the format of glucose monitoring smart sensors, commercial products are developed with biosensors, such as the wearable device that performs collection of reverse iontophoresis glucose with watch functions [115]. However, one problem is the bacterial contamination risk, which is possible due to its size and adherence [115]. In this principle, monitoring is conducted with the fusion technique in a smart sensor worn like a bracelet [169]. Two accelerometers are used to obtain data related to the arm and body motion; thermistors measure temperature of the body; a heat flux sensor measures heat loss on the skin; a galvanic sensor measures the conductivity of the subject; and ECG electrodes measure the respiratory rate and QRS complex signals; where QRS complex signal is the combination of three of the graphical deflections seen on a typical electrocardiogram signal (ECG).

**Figure 13:**
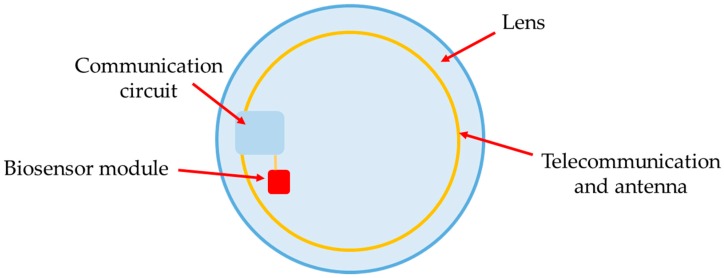
Smart sensor for glucose level measurement developed into a contact lens.

Besides wearables, smart sensors may be applied to other parts of the body, such as contact lenses used for glucose measurement through lacrimal fluid [170], shown in Figure 13. The measurement is made in a polymer lens that includes a module with three electrodes (biosensors) and a communication module, developed with microelectronic components and with a coil around the lens functioning as an antenna for data communication.

For monitoring patients with Parkinson’s disease, systems use inertial sensors mostly. This fact is justified by Parkinson’s being a neurodegenerative disease [171], which has symptoms like tremors in the hands, arms, legs and face, the stiffness of the limbs, bradykinesia (slowness to perform movements [172]), and postural instability [173]. This disease affects about 6.3 million people around the world [174].

The fusion of data from three accelerometers in an acquisition system [175] used in a device [176], provides data regarding the possible risks of the patient to fall, with less response time and accuracy in relation to other analysis, such as fall history, gait analysis, and gait locking (freezing) [175]. In the wearable field, accelerometers and vibration and force sensors are used as smart sensors applied in tracksuits [177,178] and gloves [179]. They are also applied to Parkinson’s and enable monitoring of the patient’s activities in a more natural way.

The freezing of gait (FOG) is a major concern for Parkinson’s patients. For this application, smart sensors are installed in headsets, which are exemplified by [171,180] for home environment monitoring [180]. Accelerometers, gyroscopes, and 3-axes magnetometers are allocated in a headset format, with recognition through a neural network to prevent catastrophic events, such as falls. The purpose of this system is to have a feedback loop [180], that occurs in [171]. With two inertial units allocated to the ankle, there is the data fusion in a smart sensor with an implemented algorithm to detect FOGs and warn the user through headsets to support the gait of the patient and reduce accidents [171]. Another proposal for monitoring patients with Parkinson’s disease occurs with the fusion of force sensors (for step detection) and respiration sensors (via inductive plethysmography). These are integrated into a network capable of providing real-time movement and breathing in a smart sensor operating in a smart network [181].

### 4.4. Other Applications

The following section presents works that, in nature, clash with the previously reported applications, either because they have specific characteristics or because they are situated in concepts that can be used in clinical systems**,** monitoring, and rehabilitation. An example of this is a smart sensor for gaseous analysis through a person exhalation [182], which can be used for diagnosis and monitoring. Another study, at an early stage, is the fusion of a galvanic response skin sensor, a temperature sensor, and a position sensor to detect epileptic seizures [183].

**Figure 14:**
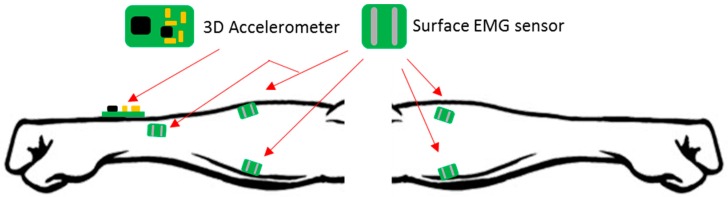
Sensor Fusion proposed using accelerometers and EMG electrodes.

Among other applications, there is recognition of movements. Its importance lies in the use of remote controls, aided by video or reproduced by robots and smart systems for interpretation. The sensors used for this are inertial, coupled with another signal that can provide additional information, such as a vision system [184]. Fusing such data results in the recognition of the movements of the hand. Accordingly, for human-computer interaction, an instrumented glove merges data from a vision system with five degrees of freedom with bending sensors based on optical fiber by means of a Kalman filter, with a 79% accuracy increase of close interphalangeal joints in relation to other systems [185]. However, another signal may be used instead of a camera, which recognizes hand movements, by using accelerometers (three-axes) and EMG signals [186]. The authors note that these tools are more accurate than those based on gloves and vision recognition [186]. Sensors were developed in the presented topology and the device is placed on the individual as shown in Figure 14. Listed total of 72 words from the Chinese symbolic language alphabet were listed.

**Figure 15:**
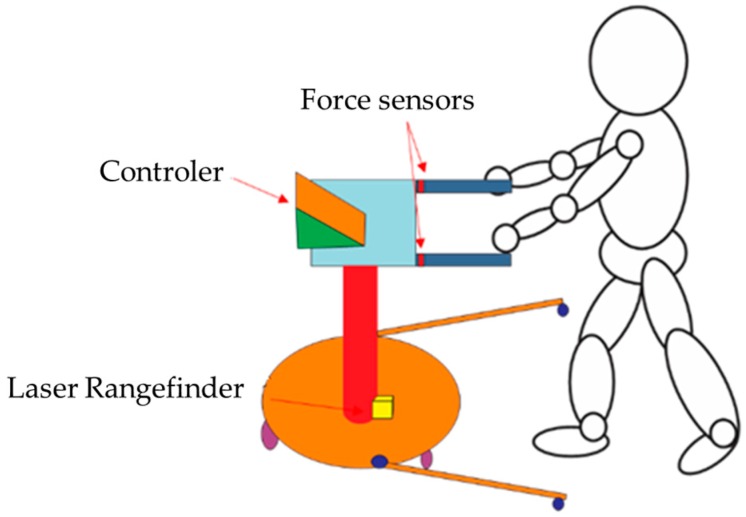
Mobile aid robot highlighting force sensors, the laser indicator, and controller.

Fusion obtained by a force sensor and laser enables the estimation of human motion [187]. Thus, a mobile robot for assistance was designed in a safe, effective, and comfortable way to monitor the coordination of arms and legs, as seen in Figure 15. These instrumented robot models help elderly people by facilitating their movements and mobility. Force sensors were based on resistive sensors, inserted in the structure as shown in Figure 15, which estimate both strength and torque in the region of the handles. The laser pointer used was attached to the bottom area of a robot, monitoring movements of the legs. Data were fused using a Kalman filter, displaying the intention of movements of the patient. A similar proposal is a robot that detects people, merging data from a camera and an RGB-D sensor in order for the patient, especially the elderly, to be traced while in their home. Their faces can be recognized by the robot and, through voice command, the user can order it to move and intervene in dangerous or risk situations [188].

Brain-Computer Interfaces (BCI) are also developed in the form of a smart sensor, with sensors compounded of EEG electrodes. In m-Health, for treatment and diagnosis of neurological disorders, a smart sensor is used with EEG electrodes with a complete processing system [189]. Based on the psychological state of a person, a smart sensor is used to change the music automatically with a BCI [190]. Moreover, systems used to merge data with BCIs containing accelerometers to control prostheses are present [191].

Exams and imaging systems [192] merge medical use data images through stationary wavelet transformation techniques and Non-Sampled Contourlet Transformed (NSCT). Both techniques improve the variance information and the fused image phase, still using Principal Component Analysis (PCA) and fusion rules to minimize redundancy, offering enhanced contrast and restoration of morphological details. To increase the functioning knowledge of the brain in normal conditions or in pathology presence, EEG fusion with functional magnetic resonance imaging is used [193], combining an analysis in space. A magnetometer and gradiometer signals can be fused to magnetoencephalography, which allows the improvement of results in a single mode [194]. For visualization of the veins, an ultrasonic sensor with a magnetic tracker is employed in the reconstruction of the arteries for arterial intraluminal prosthesis stent allocation.

## 5. Conclusions 

This work presented a different approach to the usage of smart sensors and sensor fusion according to the application (sports and biomedical). In this case, it became clear that the applications could be used as a support to the start and development of new projects in both areas. 

To formulate this review, technical repositories and references were used, preferably from the last six years. Among these references, there is a three-level classification: papers published in journals, transactions, magazines, and others technical journals from 2010 to the present (48%); papers on technical conferences, proceedings, annals, and symposiums (38%); and the remaining (14%) are general references to conceptualization.

The use of two or more sensors allows better solutions to the problems, which cannot be solved with only one type of data. The applications of smart systems coupled with processing and transmission data grant integration of data from multiple devices. The devices that use sensor fusion and smart sensors techniques are more complex than usual devices. Ergo, the use of these techniques provides a new procedure to acquire, process and transmit the same data with a different approach and innovative analysis. 

In the second context, attention was given to the growing demand in biomedical and sports applications. This demand is driven by the rapid development of electronics, allowing the construction of sensors and controllers with reduced size, low cost and high reliability. Therefore, by assessing the different applications, it is remarkable that these two areas have much more similarities than differences.

In the sports industry, sports equipment and the instrumentation of athletes presents better conditions so that sports assessment and athletic performance can be improved. Within the sports environment, training load quantification and evaluation of physical, physiological and technical conditions are of paramount importance for the development of the athlete and injury prevention. In order to perform these measurements, smart sensors, and sensor fusion provide numerical, graphical and temporal analysis, offering feedback that can be the basis for decision-making. This work presents smart sensors and sensor fusion application for the following sports: individual, collective, those with safety equipment, those in need of an assistance of an arbitrator, among others; all of which evolve by using these techniques. Thus, the importance of introducing such technology for understanding and developing sports is evident.

The execution of activities in sports is mostly dynamic. Given this premise, the use of smart sensors and sensor fusion enables data collection to be performed in real proof conditions, rather than only in the laboratory. This change of environment, when assessing in real time, demands reliable analysis that shows the performance of the athlete while performing his/her sport. With this new perspective of evaluation and monitoring of athletes, their evolution of various characteristics has become measurable, enabling more complex assessments both in real-time and in future analysis.

On the other hand, most biomedical applications present their use in concentrated or in special environments, such as hospitals and clinics. These applications relied on expensive and difficult operation/handling equipment, such as large imaging equipment. However, also boosted by the electronic development, many of the applications left the vicinity of medical centers and became part of the home, thanks to aid equipment and remote measurement development (fostered by technologies such as m-health, homecare). In this context, the use of techniques of sensor fusion and smart sensors, as seen throughout the article, has established mobile, practical, and feasible solutions with great expansion potential.

It is apparent that many applications that use techniques discussed in this article are between these two branches (biomedical and sports instrumentation). The difficulty of separating the applications for these two areas is evident when the same smart sensor or sensor fusion can be designed for both areas. For example, the heart rate monitored by a smart sensor can be applied to load training control, as well as to homecare. Thus, Section 3, which presented cases of applications that pervade both classes, is appropriate.

The main purpose was to show the interaction between sports and biomedical applications that operated with smart sensors, sensor fusion or both in the same system. The introduction of these techniques helped to understand the human body and its activities; moreover, how it was determinant to the evolution of the aforementioned areas. Finally, this type of review was necessary to bring together similar areas and collaborate with studies following the evolution of this technology.

## Figures and Tables

**Table 1:** General applications for sensor fusion and smart sensor on sports.

Sport	Type of Analysis
Alpine Skiing [75]	Movement and techniques
Tennis [76,77]	Swing and rules (challenge)
Snowboard [78,79]	Real-Time feedback of snowboarding
Martial Arts (general) [80,81]	Movement and technics
Taekwondo [80,82,83]	Movement, technics and rules (system)
General Sports [84,85]	Classification of the modality or activity of the sport

## References

[1] Luo C.R., Chang C.C., Lai C.C. (2011). Multisensor fusion and integration: Theories, applications, and its perspectives. IEEE Sens. J..

[2] Takahashi K., Yamasaki H., Irwin D. (1997). Audio-Visual Sensor Fusion System for Intelligent Sound Sensing. The Industrial Electronics Handbook.

[3] Luo C.R., Chang C.-C. (2012). Multisensor fusion and integration: A review on approaches and its applications in mechatronics. IEEE Trans. Ind. Inf..

[4] Aziz A.M. (2014). A New Adaptive Decentralized Soft Decision Combining Rule for Distributed Sensor Systems with Data Fusion. Inf. Sci..

[5] Dasarathy B.V. (1997). Sensor Fusion Potential Exploitation—Innovative Architectures and Illustrative Applications. IEEE Proc..

[6] 1451.5-2007-IEEE Standard for a Smart Transducer Interface for Sensors and Actuators Wireless Communication Protocols and Transducer Electronic Data Sheet (TEDS) Formats. http://ieeexplore.ieee.org/stamp/stamp.jsp?tp=&arnumber=4346346.

[7] Chaudhari M., Draravath S. (2014). Study of Smart Sensors and their Applications. Int. J. Adv. Res. Comput. Commun. Eng..

[8] Yurish S.Y., Yurish S.Y., Gomes M.T.S.R. (2003). Smart sensors for electrical and non-electrical, physical and chemical variables: State of the art. Smart Sensors and MEMS.

[9] Frank R. (2000). Understanding Smart Sensors.

[10] Singh V.R. (2005). Smart sensors: Physics, technology and applications. Indian J. Pure Appl. Phys..

[11] Magno M., Benini L., Gaggero L., La Torre Aro J.P., Popovici E. A versatile biomedical wireless sensor node with novel drysurface sensors and energy efficient power management. Proceedings of the 5th IEEE International Workshop on Advances in Sensors and Interfaces.

[12] Harms H., Amft O., Winkler R., Schumm J., Kusserow M., Troester G. ETHOS: Miniature orientation sensor for wearable human motion analysis. Proceedings of the 2010 IEEE Sensors.

[13] Hunter A., Coggan A.R. (2010). Training and Racing with a Power Meter.

[14] Siirtola P., Laurinen P., Röning J., Kinnunen H. Efficient accelerometer-based swimming exercise tracking. Proceedings of the 2011 IEEE Symposium on Computational Intelligence and Data Mining.

[15] Zagatto A.M., Beck W.R., Gobatto C. (2009). Vality of the Running Anaerobic Sprint Test for Assessing Anaerobic Power Predicting Short-Distance Performance. J. Strength Cond. Res..

[16] Ishido H., Takahashi H., Nakai A., Takahata T., Matsumoto K., Shimoyama I. 6-Axis force/torque sensor for spike pins of sports shoes. Proceedings of the 2015 8th IEEE International Conference on Micro Electro Mechanical Systems.

[17] Rodrigues J.R., Craveiro W.A., Lemos T.V., Passos F.A.G., de Macedo O.G., Matheus J.P.C. (2014). Influence of application of the inelastic taping in plantar pressure of runners pronators. Man. Ther. Posturol. Rehabil. J..

[18] Jung P.-G., Lim G., Kong K. A Mobile Motion Capture System Based On Inertial Sensors and Smart Shoes. Proceedings of the 2013 IEEE International Conference on Robotics and Automation.

[19] Xu J.Y., Nan X., Ebken V., Wang Y., Pottie G.J., Kaiser W.J. (2015). Integrated Inertial Sensors and Mobile Computing for Real-Time Cycling Performance Guidance via Pedaling Profile Classification. IEEE J. Biomed. Health Inf..

[20] Fuss F.K., Fuss F.K., Subic A., Strangwood M., Mehta R. (2014). Instrumentation Technology: Instrumentation of Sports Equipment. Routledge Handbook of Sports Technology and Engineering.

[21] Azcueta J.P.V., Libatique N.C., Tangonan G.L. In situ sports performance analysis system using inertial measurement units, high-fps video camera, and the Android platform. Proceedings of the 2014 International Conference on Humanoid, Nanotechnology, Information Technology, Communication and Control Environment and Management.

[22] Kaiut J.P., da Silva A.I., do Nascimento A.J. (2014). Análise do desempenho dos atletas nas provas combinadas no período de 2000 a 2012. Rev. Bras. Prescr. Fisiol. Exerc..

[23] Senanayake C., Senanayake S.M.N.A. Human assisted tools for gait analysis and intelligent gait phase detection. Proceedings of the 2009 Innovative Technologies in Intelligent Systems and Industrial Applications.

[24] Fei X., Mo P., Liu G. Development of foot surface pressure distribution measurement system for the training of soccer players. Proceedings of the 2013 Intelligent Control and Information Processing.

[25] Sobral H., Vieira A., Ferreira J.P., Ferreira P., Cruz S., Crisóstomo M., Coimbra A.P. Human gait analysis using instrumented shoes. Proceedings of the 2015 IEEE 4th Portuguese Meeting on Bioengineering.

[26] Sun N., Liu X., Wu X., Wang H. Human gait modeling and gait analysis based on Kinect. Proceedings of the 2014 IEEE International Conference on Robotics and Automation.

[27] Nukala B., Shibuya N., Rodriguez A., Tsay J., Nguyen T., Zupanic S., Lie D.Y.C. Comparing nape vs. T4 placement for a mobile Wireless Gait Analysis sensor using the Dynamic Gait Index test. Proceedings of the 2015 Eighth International Conference on Mobile Computing and Ubiquitous Networking.

[28] Salarian A., Burkhard P.R., Vingerhoets F.J., Jolles B.M., Aminian K. (2013). A novel approach to reducing number of sensing units for wearable gait analysis systems. IEEE Trans. Biomed. Eng..

[29] Wang B., Rajput K.S., Tam W.-K., Tung A.K.H., Yang Z. FreeWalker: A smart insole for longitudinal gait analysis. Proceedings of the 2015 37th Annual International Conference of the IEEE Engineering in Medicine and Biology Society.

[30] De Lima J.J., Martins M.S.R., Schleder J.C., Okida S., Stevan S.L. (2013). Dispositivo para análise dinâmica da marcha humana utilizando sensores inerciais MEMS. Rev. Engenharia Tecnol..

[31] International Association of Athletics Federations. http://www.iaaf.org/disciplines.

[32] Daukantas S., Marozas V., Lukosevicitus A. Inertial sensor for objective evaluation of swimmer performance. Proceedings of the 2008 11th International Biennial Baltic Electronics Conference.

[33] Sheaffer D.A., Burnett D.C. Improved surface swimmer detection through multimodal data fusion. Proceedings of the 2012 IEEE International Carnahan Conference on Security Technology.

[34] De Magalhaes F.A., Vannozzi G., Gatta G., Fantozzi S. (2014). Wearable inertial sensors in swimming motion analysis: A systematic review. J. Sports Sci..

[35] Rowlands D.D., James D.A., Lee J.B. (2013). Visualization of wearable sensor data during swimming for performance analysis. Sport Technol..

[36] Gong M., Zhang L., Ding Z., Dong F., Wang L. Research and development of swimming training information system based on ZigBee technology. Proceedings of the 2012 International Conference on Systems and Informatics.

[37] Khoo B.H., Lee K.J., Senanayake S.M.N.A., Wilson B.D. System for determining within-stroke variations of speed in swimming (SWiSS). Proceedings of the 2009 IEEE/ASME International Conference on Advanced Intelligent Mechatronics.

[38] Dadashi F., Crettenand F., Millet G., Seifert L., Komar J., Aminian K. (2011). Frontcrawl propulsive phase detection using inertial sensors. Port. J. Sport Sci..

[39] Carneiro D.D.A., Stevan S.L. Sistema microcontrolado para treinos de natação com interface Android. Proceedings of the Conferência Ibero Americana em Computação Aplicada.

[40] Chakravorti N., Sage T.L., Slawson S.E., Conway P.P., West A.A. (2013). Design and Implementation of an Integrated Performance Monitoring Tool for Swimming to Extract Stroke Information at Real Time. IEEE Trans. Hum. Mach. Syst..

[41] Ride J., Ringuet C., Rowlands D., Lee J., James D. (2013). A Sports Technology Needs Assessment for Performance Monitoring in Swimming. Proc. Eng..

[42] Babayan J., Hommaid M., Hage-Diab A., Abdulnabi S. Low-cost dry swimming machine using Kinect biomotion capture. Proceedings of the Low-Cost Dry Swimming Machine Using Kinect Biomotion Capture.

[43] Hagem R.M., Thiel D.V., O’Keefee S., Fickenscher T. (2013). Real-time swimmers’ feedback based on smart infrared (SSIR) optical wireless sensor. Electron. Lett..

[44] Bächlin M., Tröster G. (2012). Swimming performance and technique evaluation with wearable acceleration sensors. Pervasive Mob. Comput..

[45] Sage T. L., Conway P., Justham L., Slawson S., Bindel A., West A. A component based integrated system for signal processing of swimming performance. Proceedings of the 2010 International Conference on Signal Processing and Multimedia Applications.

[46] Hagem R.M., Thiel D.V., O’Keefe S.G., Dahm N., Stamm A., Fickenscher T. Smart optical wireless sensor for real time swimmers feedback. Proceedings of the 2012 IEEE Sensors.

[47] Gharghan S.K., Nordin R., Ismail M. Empirical investigation of pedal power calculation techniques for track cycling performance measurement. Proceedings of the 2013 IEEE Student Conference on Research and Development.

[48] Friel J. (2012). The Power Meter Handbook: A User’s Guide for Cyclists and Triathletes.

[49] Zhang Y., Beenakker K.G.M., Butala P.M., Lin C.-C., Little T.D.C., Maier A.B., Stijntjes M., Vartanian R., Wagenaar R.C. Monitoring walking and cycling of middle-aged to older community dwellers using wireless wearable accelerometers. Proceedings of the 2012 Annual International Conference of the IEEE Engineering in Medicine and Biology Society.

[50] Cockcroft J., Muller J.H., Scheffer C. (2014). A Novel Complementary Filter for Tracking Hip Angles during Cycling Using Wireless Inertial Sensors and Dynamic Acceleration Estimation. IEEE Sens. J..

[51] Zhang Y., Chen K., Yi J. (2013). Rider Trunk and Bicycle Pose Estimation with Fusion of Force/Inertial Sensors. IEEE Trans. Biomed. Eng..

[52] Neto W.R. (2013). Automatic arbitration to help a referee in soccer matches. Rev. Technoeng..

[53] Shan P., Muchhala R., Shan G. (2014). A Review Paper on Goal-Line Technology. Int. J. Curr. Eng. Technol..

[54] Maarslet H.P. (2014). Goal Detector for Detection of an Object Passing a Goal Plane. U.S. Patent.

[55] Psiuk R., Seidl T., Strauß W., Bernhard J. (2014). Analysis of Goal Line Technology from the Perspective of an Electromagnetic Field based Approach. Proc. Eng..

[56] Wakabayashi S., Ashida K., Todoroki S., Koide K. Development of a multi-purpose display and a timer for athletics. Proceedings of the 2014 IEEE 3rd Global Conference on Consumer Electronics.

[57] Redvka P.E. (2014). Estudo Correlacional Entre Variáveis Fisiológicas e da Composição Corporal com a Demanda de Movimentação e Velocidades de Deslocamento durante o Jogo de Futebol. Master’s Thesis.

[58] Rupcic T., Knjaz D., Bakovic M., Devrnja A., Matkovic B.R. (2015). Impact of fatigue on accuracy and changes in certain kinematic parameters during shooting in basketball. Hrvatski Športskomedicinski Vjesnik.

[59] Taniguchi A., Watanabe K., Kurihara Y. Measurement and analyze of jump shoot motion in basketball using a 3-D acceleration and gyroscopic sensor. Proceedings of the SICE Annual Conference.

[60] Abdelrasoul E., Mahmoud I., Stergiou P., Katz L. (2015). The Accuracy of a Real Time Sensor in an Instrumented Basketball. Proc. Eng..

[61] Toledo L.C.S. (2014). Avaliação das Variáveis Técnicas no Contexto Esportivo. Bachelor’s Thesis.

[62] Alwadi A.M.H.S. (2014). Collision Monitoring and Alarm in Ice-Hokey. Master’s Thesis.

[63] Crisco J.J., Fiore R., Beckwith J.G., Chu J.J., Brolinson P.G., Duma S., McAllister T.W., Duhaime A.C., Greenwald R.W. (2010). Frequency and location of head impact exposures in individual collegiate football players. J. Athl. Train..

[64] Mihalik J.P., Guskiewicz K.M., Marshall S.W., Blackburn J.T., Cantu R.C., Greenwald R.W. (2012). Head impact biomechanics in youth hockey: Comparisons across playing position, event types, and impact locations. Ann. Biomed. Eng..

[65] Crisco J.J., Wilcox B.J., Beckwith J.G., Chu J.J., Duhaime A.C., Maerlender A.C., McAllister T.W., Greenwald R.M. (2011). Head impact exposure in collegiate football players. J. Biomech..

[66] Wilcox B.J., Beckwith J.G., Greenwald R.W., Chu J.J., McAllister T.W., Flashman L.A., Maerlender A.C., Duhaime A.C., Crisco J.J. (2013). Head impact exposure in male and female collegiate ice hockey players. J. Biomech..

[67] Beckwith J.G., Greenwald R.M., Chu J.J., Crisco J.J., Rowson S., Duma S.M., Broglio S.P., McAllister T.W., Guskiewicz K.M., Mihalik J.P. (2013). Head Impact Exposure Sustained by Football Players on Days of Diagnosed Concussion. Sports Exerc..

[68] Daniel R.W., Rowson S., Duma S.M. (2012). Head Impact Exposure in Youth Football. Ann. Biomed. Eng..

[69] Greenwald R.M., Gwin J.T., Chu J.J., Crisco J.J. (2008). Head impact severity measures for evaluating mild traumatic brain injury risk exposure. Neurosurgery.

[70] Crisco J.J., Wilcox B.J., Machan J.T., McAllister T.W., Duhaime A.C., Duma S.M., Rowson S., Beckwith J.G., Chu J.J., Greenwald R.M. (2012). Magnitude of Head Impact Exposures in Individual Collegiate Football Players. J. Appl. Biomechan..

[71] Mertz L. (2013). Making Sports Safer for Kids: Using Biomechanical Devices to Prevent Injuries. IEEE Pulse.

[72] Hardegger M., Ledergerber B., Mutter S., Vogt C., Seiter J., Calatroni A., Tröster G. Sensor Technology for Ice Hockey and Skating. Proceedings of the IEEE 12th International Conference on Wearable and Implantable Body Sensor Networks.

[73] Vales-Alonso J., Chavez-Diéguez D., Lópes-Matencio P., Acaraz J.J., Parrado-García F.J., González-Castaño J. (2015). SAETA: A Smart Coaching Assistant for Professional Volleyball Training. IEEE Trans. Syst. Man Cybern. Syst..

[74] Can Y.S., Dönmez M.Y. Sport Sense: A mobile sensor data collection, labeling and display application for sport centers. Proceedings of the 2015 23rd Signal Processing and Communications Applications Conference.

[75] Supej M. (2010). 3D measurements of alpine skiing with an inertial sensor motion capture suit and GNSS RTK system. J. Sports Sci..

[76] Takano K., Li K.F. (2013). A multimedia tennis instruction system: Tracking and classifying swing motions. Int. J. Space-Based Situat. Comput..

[77] Clarke S.R., Norman J.M. (2012). Optimal challenges in tennis. J. Oper. Res. Soc..

[78] Spelmezan D., Borchers J. Real-time Snowboard Training System. Proceedings of the Conference on Human Factors in Computing Systems.

[79] Holleczek T., Rüegg A., Harms H., Tröster G. Textile pressure sensors for sports applications. Proceedings of the 2010 IEEE Sensors.

[80] Chi E.H. (2005). Introducing Wearable Force Sensors in Martial Arts. IEEE Pervasive Comput..

[81] Darius D.D.I., Ridzuan S.J., Deros B.M., Ramli A.S. Female student-athletes’ biomechanics and anthropometric profile of unarmed combat kicks. Proceedings of the 2014 IEEE Conference on Biomedical Engineering and Sciences.

[82] Lee S.-B., Cha E.-J., Lee T.-S. Analysis of physical activities in Taekwondo Pumsae. Proceedings of the 2008 30th Annual International Conference of the IEEE Engineering in Medicine and Biology Society.

[83] Peng L., Yaping Z. The Design and Realization of the Taekwondo Real-Time Hit Effect and Feedback System. Proceedings of the 2015 2nd International Conference on Information Science and Control Engineering.

[84] Cricri F., Roininen M., Mate S., Leppännen J., Curcio I.D.D., Gabbouj M. Multi-sensor fusion for sport genre classification of user generated mobile videos. Proceedings of the 2013 IEEE International Conference on Multimedia and Expo.

[85] Liu S., Gao R.X., John D., Staudenmayer J.W., Freedson P.S. (2012). Multisensor Data Fusion for Physical Activity Assessment. IEEE Trans. Biomed. Eng..

[86] Razak A.H.A., Zayegh A., Begg R.K., Wahab Y. (2012). Foot Plantar Pressure Measurement System: A Review. Sensors.

[87] Manupibul U., Charoensuk W., Kaimuk P. Design and development of SMART insole system for plantar pressure measurement in imbalance human body and heavy activities. Proceedings of the 2014 7th Biomedical Engineering International Conference.

[88] Wafai L., Zayegh A., Woulfe J., Aziz S.M., Begg R. (2015). Identification of Foot Pathologies Based on Plantar Pressure Asymmetry. Sensors.

[89] Catalfamo P., Moser D., Ghoussayni S., Ewins D. (2008). Detection of gait events using an F-Scan in-shoe pressure measurement system. Gait Posture.

[90] Loss J.F., Cantergi D., Krumholz F.M., Torre M.L., Conditti C.T. (2013). Evaluating the Electromyographical Signal during Symmetrical Load Lifting. Braz. J. Oral Sci..

[91] Holmberg P.M. (2013). Weightlifting to Improve Volleyball Performance. Strength Cond. J..

[92] Willick S.E., Cuschman D., Blauwet C.A., Emery C., Webborn N., Derman W., Schwellnis M., Stomphorst J., de Vliet P.V. (2015). The epidemiology of injuries in powerlifting at the London 2012 Paralympic Games: An analysis of 1411 athlete-days. Scand. J. Med. Sci. Sports.

[93] Tao W., Lui T., Zheng R., Feng H. (2012). Gait Analysis Using Wearable Sensors. Sensors.

[94] Curone D., Tognetti A., Secco E.L., Anania G., Carbonaro N., De Rossi D., Magenes G. (2010). Heart Rate and Accelerometer Data Fusion for Activity Assessment of Rescuers During Emergency Interventions. IEEE Trans. Inf. Technol. Biomed..

[95] Tang Z., Sekine M., Tamura T., Tanaka N., Yoshida M., Chen W. (2015). Measurement and Estimation of 3D Orientation using Magnetic and Inertial Sensors. Adv. Biomed. Eng..

[96] Alahakone A.U., Senanayake S.M.N.A. (2010). A Real-Time System with Assistive Feedback for Postural Control in Rehabilitation. IEEE/ASME Trans. Mechatron..

[97] Sardini E., Serpelloni M., Pasqui V. (2015). Wireless Wearable T-Shirt for Posture Monitoring During Rehabilitation Exercises. IEEE Trans. Instrum. Meas..

[98] Hong Y.-J., Kim I.-J., Ahn S.C., Kim H.-G. (2010). Mobile health monitoring system based on activity recognition using accelerometer. Simul. Model. Pract. Theor..

[99] Lu Y., Huang J., Xu W., Tao C., Wang X. An Electronic Travel Aid based on multi-sensor fusion using extended Kalman filter. Proceedings of the 2014 11th World Congress on Intelligent Control and Automation.

[100] Tognetti A., Lorussi F., Carbonaro N., de Rossi D. (2015). Wearable Goniometer and Accelerometer Sensory Fusion for Knee Joint Angle Measurement in Daily Life. Sensors.

[101] Salarian A., Horak F.B., Zampiere C., Carlson-Kuhta P., Nutt J.G., Aminian K. (2010). iTUG, a sensitive and reliable measure of mobility. IEEE Trans. Neural Syst. Rehabil. Eng..

[102] Mcllwraith D., Pansiot J., Yang G.-Z. Wearable and ambient sensor fusion for the characterisation of human motion. Proceedings of the 2010 IEEE/RSJ International Conference on Intelligent Robots and Systems.

[103] Majoe D., Bonhof P., Kaegi-Trachsel T., Gutknecht J., Widmer L. Stress and sleep quality estimation from a smart wearable sensor. Proceedings of the 2010 5th International Conference on Pervasive Computing and Applications.

[104] Bartalesi R., Lorussi F., De Rossi D., Tesconi M., Tognetti A. Wearable monitoring of lumbar spine curvature by inertial and e-textile sensory fusion. Proceedings of the 2010 Annual International Conference of the IEEE Engineering in Medicine and Biology.

[105] Antink C.H., Brüser C., Leonhardt S. Multimodal sensor fusion of cardiac signals via blind deconvolution: A source-filter approach. Proceedings of the Computing in Cardiology 2014.

[106] Lahat D., Adali T., Jutten C. (2015). Multimodal Data Fusion: An Overview of Methods, Challenges, and Prospects. IEEE Proc..

[107] Patel S., Park H., Bonato P., Chan L., Rodgers M. (2012). A review of wearable sensors and systems with application in rehabilitation. J. Neuroeng. Rehabil..

[108] Potluri C., Anugolu M., Schoen M.P., Naidu D.S., Urfer A., Rieger C. Computational intelligence based data fusion algorithm for dynamic sEMG and skeletal muscle force modelling. Proceedings of the 2013 6th International Symposium on Resilient Control Systems.

[109] Murai R., Sakai T., Kitano Y., Honda Y. Recognition of 3D dynamic environments for mobile robot by selective memory intake and release of data from 2D sensors. Proceedings of the 2012 IEEE/SICE International Symposium on System Integration.

[110] Moslem B., Khalil M., Diab M.O., Marque C. Classification of multichannel uterine EMG signals by using a weighted majority voting decision fusion rule. Proceedings of the 2012 16th IEEE Mediterranean Electrotechnical Conference.

[111] González F.C., Villegas O.O., Ramiréz D.E., Sánchez V.G., Domínguez H.O. (2014). Smart multi-level tool for remote patient monitoring based on a wireless sensor network and mobile augmented reality. Sensor.

[112] De Capua C., Meduri A., Morello R. (2010). A Smart ECG Measurement System Based on Web-Service-Oriented Architecture for Telemedicine Applications. IEEE Trans. Instrum. Meas..

[113] Venema B., Schiefer J., Blazek V., Blanik N., Leonhardt S. (2013). Evaluating Innovative In-Ear Pulse Oximetry for Unobtrusive Cardiovascular and Pulmonary Monitoring During Sleep. IEEE J. Transl. Eng. Health Med..

[114] Morello R. (2015). Use of TEDS to Improve Performance of Smart Biomedical Sensors and Instrumentation. IEEE Sens..

[115] Ponmozhi J., Frias C., Marques T., Frazão O. (2012). Smart sensors/actuators for biomedical applications: Review. Measurements.

[116] Massot B., Risset T., Micheletm G., McAdams E. A wireless, low-power, smart sensor of cardiac activity for clinical remote monitoring. Proceedings of the 2015 17th International Conference on E-health Networking, Application & Services.

[117] Kelly S.K., Shire D.B., Chen J., Doyle P., Gingerich M.D., Cogan S.F., Drohan W.A., Behan S., Theogarajan L., Wyatt J.L. (2011). A Hermetic Wireless Subretinal Neurostimulator for Vision Prostheses. IEEE Trans. Biomed. Eng..

[118] Harsányi G. (2000). Sensors in Biomedical Applications: Fundamentals, Technology and Applications.

[119] Trigno ^TM^ Wireless EMG. Delsys, Wearable Sensors for Movement Sciences. http://www.delsys.com/products/wireless-emg/.

[120] Mamun K.A., Sharma A., Hoque A.S.M., Szecsi T. Remote patient physical condition monitoring service module for iWARD hospital robots. Proceedings of the 2014 Asia-Pacific World Congress on Computer Science and Engineering.

[121] Sardino E., Serpelloni M. (2013). T-Shirt for Vital Parameter Monitoring. Lect. Notes Electr. Eng..

[122] Kalatunga T.N., Ranasinghe R.A.G.P., Ranathunga R.A.C., Ratnayake R.A.C.H., Nanayakkara N.D. Real time endoscope trajectory tracking in the 3D space using MEMS sensors. Proceedings of the 2013 IEEE 8th International Conference on Industrial and Information Systems.

[123] Mauro E.D., Solbiati M., Beni S.D., Forzoni L., D’Onofrio S., Solbiati L. Virtual navigator real-time ultrasound fusion imaging with positron emission tomography for liver interventions. Proceedings of the 2013 35th Annual International Conference of the IEEE Engineering in Medicine and Biology Society.

[124] Appelbaum L., Solbiati L., Sosna J., Nissenbaum Y., Greenbaum N., Goldberg S.N. (2013). Evaluation of an electromagnetic image-fusion navigation system for biopsy of small lesions: assessment of accuracy in an in vivo swine model. Acad. Radiol..

[125] Honrado C., Dong T. (2014). A Capacitive Touch Screen Sensor for Detection of Urinary Tract Infections in Portable Biomedical Devices. Sensors.

[126] Dwyer G., Giataganas P., Pratt P., Hughes M., Yang G.-Z. A Miniaturised Robotic Probe for Real-Time Intraoperative Fusion of Ultrasound and Endomicroscopy. Proceedings of the IEEE International Conference on Robotics and Automation.

[127] Facchinetti A., Sparacino G., Guerra S., Luijf Y.M., DeVries J.H., Mader J.K., Ellmerer M., Benesch C., Heinemann L., Bruttomesso D. (2013). Real-time improvement of continuous glucose monitoring accuracy: The smart sensor concept. Diabetes Care.

[128] Ahmadi M., Rajamani R., Timm G., Sezen A.S. (2015). Flexible Distributed Pressure Sensing Strip for a Urethral Catheter. J. Microelectromech. Syst..

[129] Rajan R., Mukkundi B.K., Bhattacharya B., Bhatt O.P. Design and development of a networked health monitoring and control system. Proceedings of the 2014 4th Interdisciplinary Engineering Design Education Conference.

[130] Bellos C., Papadopoulos A., Rosso R., Fotiadis D.I. Categorization of patients’ health status in COPD disease using a wearable platform and random forests methodology. Proceedings of the 2012 IEEE-EMBS International Conference on Biomedical and Health Informatics.

[131] Rehabilitation. The Free Disctionary by Farlex. http://medical-dictionary.thefreedictionary.com/rehabilitation.

[132] Hondori H.M., Khademi M., Lopes C.V. Monitoring Intake Gestures using Sensor Fusion (Microsoft Kinect and Inertial Sensors) for Smart Home Tele-Rehab Setting. Proceedings of the 1st Annual IEEE Healthcare Innovation Conference.

[133] Senanayake S.M.N., Malik O.A., Iskandar M., Zaheer D. 3-D kinematics and neuromuscular signals’ integration for post ACL reconstruction recovery assessment. Proceedings of the 2013 35th Annual International Conference of the IEEE Engineering in Medicine and Biology Society.

[134] Kundu A.S., Mazumder O., Chattaraj R., Bhaumik S., Lenka P.K. Trajectory generation for myoelectrically controlled lower limb active knee exoskeleton. Proceedings of the 2014 Seventh International Conference on Contemporary Computing.

[135] Spulber I., Papi E., Chen Y.M., Anaastasona-Ivanova S., Bergmann J., Georgiou P., McGregor A.H. Development of a wireless multi-functional body sensing platform for smart garment integration. Proceedings of 2014 IEEE Biomedical Circuits and Systems Conference.

[136] Novak D., Reiner R. (2015). A survey of sensor fusion methods in wearable robotics. Robot. Auton. Syst..

[137] Martin H., Donaw J., Kelly R., Jung Y.J., Kim J.-H. A Novel Approach of Prosthetic Arm Control using Computer Vision, Biosignals, and Motion Capture. Proceedings of the 2014 IEEE Symposium on Computational Intelligence in Robotic Rehabilitation and Assistive Technologies.

[138] Gallego J.A., Ibanez J., Dideriksen J.L., Serrano J.I., Castillo M.D., Farina D., Rocon E. (2012). A Multimodal Human–Robot Interface to Drive a Neuroprosthesis for Tremor Management. IEEE Trans. Syst. Man Cybern..

[139] Blanca D., Kyriacou T., Lambrecht J.M., Chadwick E.K. (2016). Feasibility of using combined EMG and kinematic signals for prosthesis control: A simulation study using a virtual reality environment. J. Electromyogr. Kinesiol..

[140] Artemiadis P.K., Kyriakopoulos K.J. (2010). A Switching Regime Model for the EMG-Based Control of a Robot Arm. IEEE Trans. Syst. Man Cybern..

[141] Guo W., Sheng X., Liu H., Zhu X. (2016). Development of a Multi-Channel Compact-Size Wireless Hybrid sEMG/NIRS Sensor System for Prosthetic Manipulation. IEEE Sens. J..

[142] Hiro N., Takenshi E., Ohno K., Tadokoro S. Developing a measurement system for improving daily lives of Cycling Wheel Chair patients. Proceedings of the 2012 SICE Annual Conference.

[143] Postolocha O.A., Girao P.M.B.S., Mendes J., Pinheiro E.C., Postolache G. (2010). Physiological Parameters Measurement Based on Wheelchair Embedded Sensors and Advanced Signal Processing. IEEE Trans. Instrum. Meas..

[144] O’Flynn B., Sanchez J.T., Angove P., Connolly J., Condell J., Curran K., Gardiner P. Novel smart sensor glove for arthritis rehabilitation. Proceedings of the 2013 IEEE International Conference on Body Sensor Networks.

[145] Mohammadi-Abdar H., Ridgel A.L., Discenzo F.M., Loparo K.A. (2015). Design and Development of a Smart Exercise Bike for Motor Rehabilitation in Individuals with Parkinson’s Disease. IEEE/ASME Trans. Mechatron..

[146] Židek K., Hošovský A., Maxim V. Real-time safety circuit based on combined MEMS sensor data for Rehabilitation device. Proceedings of the 2012 13th International Carpathian Control Conference.

[147] Malciuca A., Stamatescu G., Popescu D., Struţu M. Integrating wireless body and ambient sensors into a hybrid femtocell network for home monitoring. Proceedings of the 2013 2nd International Conference on Systems and Computer Science.

[148] Olivares A., Olivares G., Mula F., Górriz J.M., Ramírez J. (2011). Wagyromag: Wireless sensor network for monitoring and processing human body movement in healthcare applications. J. Syst. Archit..

[149] Varma D., Shete V.V., Somani S.B. (2015). Development of Home Health Care Self Monitoring System. Int. J. Adv. Res. Comput. Commun. Eng..

[150] Megalingam R.K., Unnikrishnan M., Radhakrishnan V., Jacob D.C. HOPE: An electronic gadget for home-bound patients and elders. Proceedings of the 2012 Annual IEEE India Conference.

[151] Huang Y.-J., Tzeng T.-H., Lin T.-W., Huang C.-W., Yen P.-W., Kuo P.-H., Lin C.-T., Lu S.-L. (2014). A Self-Powered CMOS Reconfigurable Multi-Sensor SoC for Biomedical Applications. IEEE J. Sol. State Circuits.

[152] Bhattacharyya M., Gruenwald W., Dusch B., Aghassi-Hagmann J., Jansen D., Reindl L. A RFID/NFC based Programmable SOC for biomedical applications. Proceedings of the 2014 International SoC Design Conference.

[153] Mukherjee S., Dolui K., Datta S.K. Patient health management system using e-health monitoring architecture. Proceedings of the 2014 IEEE International Advance Computing Conference.

[154] Cheng J.-F., Chou J.-C., Sun T.-P., Hsiung S.-K., Kao H.-L. (2012). System for Monitoring of Blood Electrolytes with Wireless Home-Care System. IEEE Sens. J..

[155] Teichmann D., Matteis D.D., Walter M., Leonhardt S. A Bendable and Wearable Cardiorespiratory Monitoring Device Fusing Two Noncontact Sensor Principles. Proceedings of the 2014 11th International Conference on Wearable and Implantable Body Sensor Networks.

[156] Jourand P., Clercq H.D., Corthout R., Puers R. Textile Integrated Breathing and ECG Monitoring System. Proceedings of the Eurosensors XXIII Conference.

[157] Jourand P., Clercq H.D., Puers R. (2010). Robust monitoring of vital signs integrated in textile. Sens. Actuators A Phys..

[158] Albright R.K., Goska B.J., Hagen T.M., Chi M.Y., Cauwenberghs G., Chiang P.Y. OLAM: A wearable, non-contact sensor for continuous heart-rate and activity monitoring. Proceedings of the 2011 Annual International Conference of the IEEE Engineering in Medicine and Biology Society.

[159] Lorussi F., Carbonaro N., de Rossi D., Paradiso R., Veltink P., Tognetti A. (2016). Wearable Textile Platform for Assessing Stroke Patient Treatment in Daily Life Conditions. Front. Bioeng. Biotechnol..

[160] Wang W.-H., Chung P.-C., Hsu Y.-L., Pai M.-C., Lin C.-W. Inertial-Sensor-Based Balance Analysis System for Patients with Alzheimer’s Disease. Proceedings of the 2013 Conference on Technologies and Applications of Artificial Intelligence.

[161] Morello R., de Capua C., Meduri A. (2010). A Wireless Measurement System for Estimation of Human Exposure to Vibration during the Use of Handheld Percussion Machines. IEEE Trans. Instrum. Meas..

[162] Morello R., de Capue C., Lamonaca F. Diagnosis of gastric disorders by non-invasive myoelectrical measurements. Proceedings of the 2013 IEEE International Instrumentation and Measurement Technology Conference.

[163] Vavrinsky E., Telek P., Donoval M., Sladek L., Daricek M., Horinek F., Donoval D. (2012). Sensor System for Wireless Bio-Signal Monitoring. Proc. Chem..

[164] Accu-Chek Blood Glucose Monitoring: The Facts about Accuracy. https://www.accu-chek.com/hcpstatic/documents/product-solutions/pe-kit/REVISED_29117_49670_routing.pdf.

[165] World Health Organization Diabetes. http://www.who.int/diabetes/facts/world_figures/en/.

[166] Merriam-Webster Diabetes. http://www.merriam-webster.com/dictionary/diabetes.

[167] Caduff A., Mueller M., Megej A., Dewarrat F., Suri R.E., Klisic J., Donath M., Zakharov P., Schaub D., Stahel W.A. (2011). Characteristics of a multisensor system for non invasive glucose monitoring with external validation and prospective evaluation. Biosens. Bioelectron..

[168] Alhawari M., Khandoker A., Mohammad B., Saleh H., Khalaf K., Al-Qutayri M., Yapici M.K., Singh S., Ismail M. Energy efficient system-on-chip architecture for non-invasive mobile monitoring of diabetics. Proceedings of the 2013 8th International Conference on Design & Technology of Integrated Systems in Nanoscale Era.

[169] Sobel S.I., Chomentowski P.J., Vyas N., Andre D., Toledo F.G. (2014). Accuracy of a Novel Noninvasive Multisensor Technology to Estimate Glucose in Diabetic Subjects During Dynamic Conditions. J. Diabetes Sci. Technol..

[170] Liao Y.-T., Yao H., Lingley A., Parviz B., Otis B.P. (2012). A 3-μW CMOS Glucose Sensor for Wireless Contact-Lens Tear Glucose Monitoring. IEEE J. Sol. State Circuits.

[171] Mazilu S., Blanke U., Hardgger M., Tröster G., Gazit E., Dorfman M., Hausdorff J.M. GaitAssist: A Wearable Assistant for Gait Training and Rehabilitation in Parkinson’s Disease. Proceedings of the 2014 IEEE International Conference on Pervasive Computing and Communication Workshops.

[172] Parkinson’s Disease Symptoms. http://www.parkinsons.org/parkinsons-symptoms.html.

[173] Parkinson’s Disease Information. http://www.parkinsons.org/.

[174] European Brain Council Parkinson’s disease Fact Sheet. http://www.europeanbraincouncil.org/pdfs/Documents/Parkinson’s%20fact%20sheet%20July%202011.pdf.

[175] Weiss A., Brozgol M., Dorfman M., Herman T., Shema S., Giladi N., Hausdorff J.M. (2013). Does the evaluation of gait quality during daily life provide insight into fall risk? A novel approach using 3-day accelerometer recordings. Neurorehabil. Neural Repair.

[176] Weiss A., Herman T., Giladi N., Hausdorff J.M. (2014). Objective assessment of fall risk in Parkinson’s disease using a body-fixed sensor worn for 3 days. PLoS ONE.

[177] Niazmand K., Tonn K., Kalaras A., Kammermeier S., Boetzel K., Mehrkens J.H., Lueth T.C. A measurement device for motion analysis of patients with Parkinson’s disease using sensor based smart clothes. Proceedings of the 2011 5th International Conference on Pervasive Computing Technologies for Healthcare (PervasiveHealth) and Workshops.

[178] Niazmand K., Jehle C., D’Angelo L.T., Lueth T.C. A new washable low-cost garment for everyday fall detection. Proceedings of the 2010 Annual International Conference of the IEEE Engineering in Medicine and Biology.

[179] Niazmand K., Tonn K., Kalaras A., Fietzek U.M., Mehrkens J.H., Lueth T.C. Quantitative evaluation of Parkinson’s disease using sensor based smart glove. Proceedings of the 2011 24th International Symposium on Computer-Based Medical Systems.

[180] Lorenzi P., Rao R., Romano G., Kita A., Serpa M., Filesi F., Bologna M., Suppa A., Berardeli A. Smart sensors for the recognition of specific human motion disorders in Parkinson’s disease. Proceedings of the 2015 6th IEEE International Workshop on Advances in Sensors and Interfaces.

[181] Ying H., Schlösser M., Schnitzer A., Schäfer T., Schläfke M.E., Leonhardt S., Schiek M. (2011). Distributed intelligent sensor network for the rehabilitation of Parkinson’s patients. IEEE Trans. Inf. Technol. Biomed..

[182] Hijazi Z., Caviglia D., Valle M., Chible H. High accuracy resistance to current circuit design for resistive gas sensor biomedical applications. Proceedings of the 2015 International Conference on Advances in Biomedical Engineering.

[183] Gouravajhala S.R., Khuon L. A multi-modality sensor platform approach to detect epileptic seizure activity. Proceedings of the 2012 38th Annual Northeast Bioengineering Conference.

[184] Liu K., Chen C., Jafari R., Kehtarnavaz N. (2014). Fusion of Inertial and Depth Sensor Data for Robust Hand Gesture Recognition. IEEE Sens. J..

[185] Arkenbout E.A., de Winter J.C.F., Breedveld P. (2015). Robust Hand Motion Tracking through Data Fusion of 5DT Data Glove and Nimble VR Kinect Camera Measurements. Sensors.

[186] Zhang X., Chen X., Li Y., Lantz V., Wang K., Yang J. (2011). A Framework for Hand Gesture Recognition Based on Accelerometer and EMG Sensors. IEEE Trans. Syst. Man Cybern. A Syst. Hum..

[187] Yan Q., Xu W., Huang J., Cao S. Laser and force sensors based human motion intent estimation algorithm for walking-aid robot. Proceedings of the 2015 IEEE International Conference on Cyber Technology in Automation, Control, and Intelligent Systems.

[188] Hu N., Bormann R., Zwölfer T., Kröse B. Multi-user identification and efficient user approaching by fusing robot and ambient sensors. Proceedings of the 2014 IEEE International Conference on Robotics and Automation.

[189] Sukumaran D., Enyi Y., Shuo S., Basu A., Zhao D., Dauwels J. A low-power, reconfigurable smart sensor system for EEG acquisition and classification. Proceedings of the 2012 IEEE Asia Pacific Conference on Circuits and Systems.

[190] Tseng K.C., Lin B.S., Wong A.M., Lin B.S. (2015). Design of a mobile brain computer interface-based smart multimedia controller. Sensors.

[191] Zhang X., Li R., Li Y. Research on brain control prosthetic hand. Proceedings of the 2014 11th International Conference on Ubiquitous Robots and Ambient Intelligence.

[192] Bhateja V., Patel H., Krishn A., Sahu A., Lay-Ekuakille A. (2015). Multimodal Medical Image Sensor Fusion Framework Using Cascade of Wavelet and Contourlet Transform Domains. IEEE Sens. J..

[193] Hansen S.T., Winkler I., Hansen L.K., Müller K.-R., Dähne S. Fusing Simultaneous EEG and fMRI Using Functional and Anatomical Information. Proceedings of the 2015 International Workshop on Pattern Recognition in NeuroImaging.

[194] Mohseni H.R., Kringelbach M.L., Woolrich M.W., Aziz T.Z., Smith P.P. A New Approach to the Fusion of EEG and MEG Signals Using the LCMV Beamformer. Proceedings of the IEEE International Conference on Acoustics, Speech, and Signal Processing.

